# FBXW7/GSK3β-mediated proline-rich 11 degradation promotes oxidative DNA damage and inhibits tumor progression in renal cell carcinoma

**DOI:** 10.7150/thno.106018

**Published:** 2025-02-03

**Authors:** Siming Chen, Kangping Xiong, Jianmin Liu, Shijie Yao, Mingxing Li, Jingtian Yu, Gang Wang, Sheng Tu, Wan Jin, Jiageng Shi, Yu Xiao, Yi Zhang, Kaiyu Qian, Lingao Ju, Xinghuan Wang

**Affiliations:** 1 Department of Urology, Zhongnan Hospital of Wuhan University, Wuhan, China.; 2 Hubei Key Laboratory of Urological Diseases, Zhongnan Hospital of Wuhan University, Wuhan, China.; 3 Department of Gynecological Oncology, Zhongnan Hospital of Wuhan University, Wuhan, China.; 4 Department of Biological Repositories, Human Genetic Resources Preservation Center of Hubei Province, Zhongnan Hospital of Wuhan University, Wuhan, China.; 5 Euler Technology, ZGC Life Sciences Park, Beijing, China.; 6 Center for Quantitative Biology, School of Life Sciences, Peking University, Beijing, China.; 7 Wuhan Research Center for Infectious Diseases and Cancer, Chinese Academy of Medical Sciences, Wuhan, China.; 8 Medical Research Institute, Frontier Science Center for Immunology and Metabolism, Taikang Center for Life and Medical Sciences, Wuhan University, Wuhan, China.

**Keywords:** Renal cell carcinoma, PRR11, FBXW7, GSK3β, AKT signaling, oxidative DNA damage.

## Abstract

**Rationale:** Renal cell carcinoma (RCC) is a highly malignant and common urological tumor. In our previous study, we reported the upregulation of PRR11 in RCC, emphasizing its important role in cell cycle regulation and apoptosis. In this follow-up study, we aim to further investigate the carcinogenic mechanism of PRR11.

**Methods:** Immunoprecipitation-mass spectrometry (IP-MS), ubiquitination assays, and *in vitro* phosphorylation assays were used to investigate the phosphorylation and ubiquitination-mediated degradation of PRR11 by FBXW7 and GSK3β. RNA-seq analysis of *PRR11* knockdown RCC cells and cellular functional assays, including flow cytometry and comet assays, were performed to explore downstream signaling pathways and regulatory functions. Mouse subcutaneous tumor, tail vein lung metastasis, and popliteal lymph node metastasis models were established to validate PRR11's role* in vivo*.

**Results:** Our results reveal that GSK3β recognizes and phosphorylates the CDC4 phosphodegron (CPD) consensus motif of PRR11, enabling FBXW7 to bind to PRR11 and catalyze its K48-linked ubiquitination and degradation. Moreover, PRR11 activates AKT signaling, which inhibits GSK3β activity. This inhibition prevents the phosphorylation of CPD motifs on PRR11, thereby obstructing FBXW7-mediated ubiquitination and degradation. The interaction between PRR11 and AKT creates a positive feedback loop that increases the level of both proteins, which ultimately accelerates RCC progression by inhibiting oxidative DNA damage.

**Conclusion:** The FBXW7/GSK3β-PRR11-AKT axis plays a pivotal role in the development of RCC by regulating oxidative DNA damage. Targeting PRR11 may be a potential therapeutic strategy for RCC.

## Introduction

Renal cell carcinoma (RCC) accounts for 4.01% of all adult malignancies and 28.48% of urinary malignancies, making it the third most prevalent cancer of the urinary system after bladder and prostate cancer 1, 2. A considerable number of patients present with advanced disease, with up to 17% already harboring distant metastases at the time of diagnosis 2. Moreover, the prognosis for patients with advanced RCC is especially poor, with an average 5-year survival rate of approximately 8% 3. Oxidative stress is a hallmark of tumor cells and is pervasive throughout tumorigenesis and progression 4. Several studies have demonstrated the critical role that oxidative DNA damage plays in the development of RCC 5, 6. However, the specific mechanism underlying oxidative DNA damage in RCC remains largely elusive, emphasizing the importance of investigating RCC mechanisms for the development of potential diagnostic and therapeutic approaches.

In our previous study, we identified proline-rich 11 (PRR11) as a poor prognostic marker for RCC, demonstrating its role in promoting RCC cell proliferation and metastasis. Additionally, PRR11 can affect cell cycle progression by inducing E2F1 protein degradation via interaction with E2F1 7. Here, we further explored its molecular mechanisms in RCC. PRR11, located on chromosome 17q22-23, encodes a protein with an N-terminal structural domain containing a proline-rich region and a C-terminal structural domain with numerous motifs 8, 9. Notably, the C-terminal domain of PRR11 harbors abundant ubiquitination and phosphorylation sites crucial for PRR11 protein function 9. Subsequent research has consistently confirmed the oncogenic role of PRR11 in various tumors, attracting because of its significance as an oncogene 10. In cutaneous squamous cell carcinoma, PRR11 promotes tumor progression by activating the EGFR signaling pathway 11. In non-small cell lung cancer, PRR11 regulates cytoskeleton-nucleoskeleton assembly and chromatin remodeling by recruiting ARP2/3 complex and can promote tumor metastasis by facilitating filamentous pseudopod formation via ARP2/3 complex 12, 13. High PRR11 expression is also strongly associated with poor prognosis in other urinary system malignancies, including prostate and bladder cancer 14, 15. However, the carcinogenic mechanism of high levels of PRR11 remains to be explored.

An essential member of the F-box family, F-box and WD repeat domain containing 7 (FBXW7), serves as the target protein recognition element of the SKP1-Cullin1-F-box (SCF) E3 ubiquitin ligase complex 16. FBXW7 has been shown to degrade many key regulators of cellular function by targeting them at cell cycle checkpoints, tumor cell proliferation, DNA damage repair, genomic instability, regulation of cell survival, and tumor microenvironment regulation 17-19. For the most part, FBXW7 binds to a substrate only when the substrate with the conserved CDC4 phosphodegron (CPD) motif is phosphorylated by one or more kinases. T/S-P-X-X-S/T is the typical sequence of CPD, in which a particular kinase phosphorylates serine or threonine residues before FBXW7 recognizes them 20. Currently, numerous investigations have verified that FBXW7 mediates the ubiquitination of numerous important tumor proteins, including NOTCH1, c-MYC, c-JUN, and CCNE1 21. Therefore, FBXW7 is currently recognized as a classical tumor suppressor protein. In addition, FBXW7 was found to be widely mutated in human malignant tumors, with a total mutation rate of nearly 6% 22. In RCC, FBXW7 overexpression induces cell apoptosis and inhibits tumor proliferation and metastasis 23. However, many important FBXW7 targets involved in tumor development remain to be characterized.

In this study, we report that FBXW7 mediates the ubiquitination and degradation of PRR11 through the GSK3β-mediated phosphorylation of PRR11 at Thr287, Ser291, Thr326, and Thr330. Furthermore, we demonstrated the important role of the AKT-GSK3β axis in preventing FBXW7-mediated PRR11 degradation. Importantly, we revealed that PRR11 affects the oxidative DNA damage process in tumors and activates the AKT pathway. This regulatory network forms a positive feedback loop that accelerates RCC development.

## Results

### GSK3β promotes PRR11 phosphorylation and ubiquitin-dependent degradation

**Figure 1:**
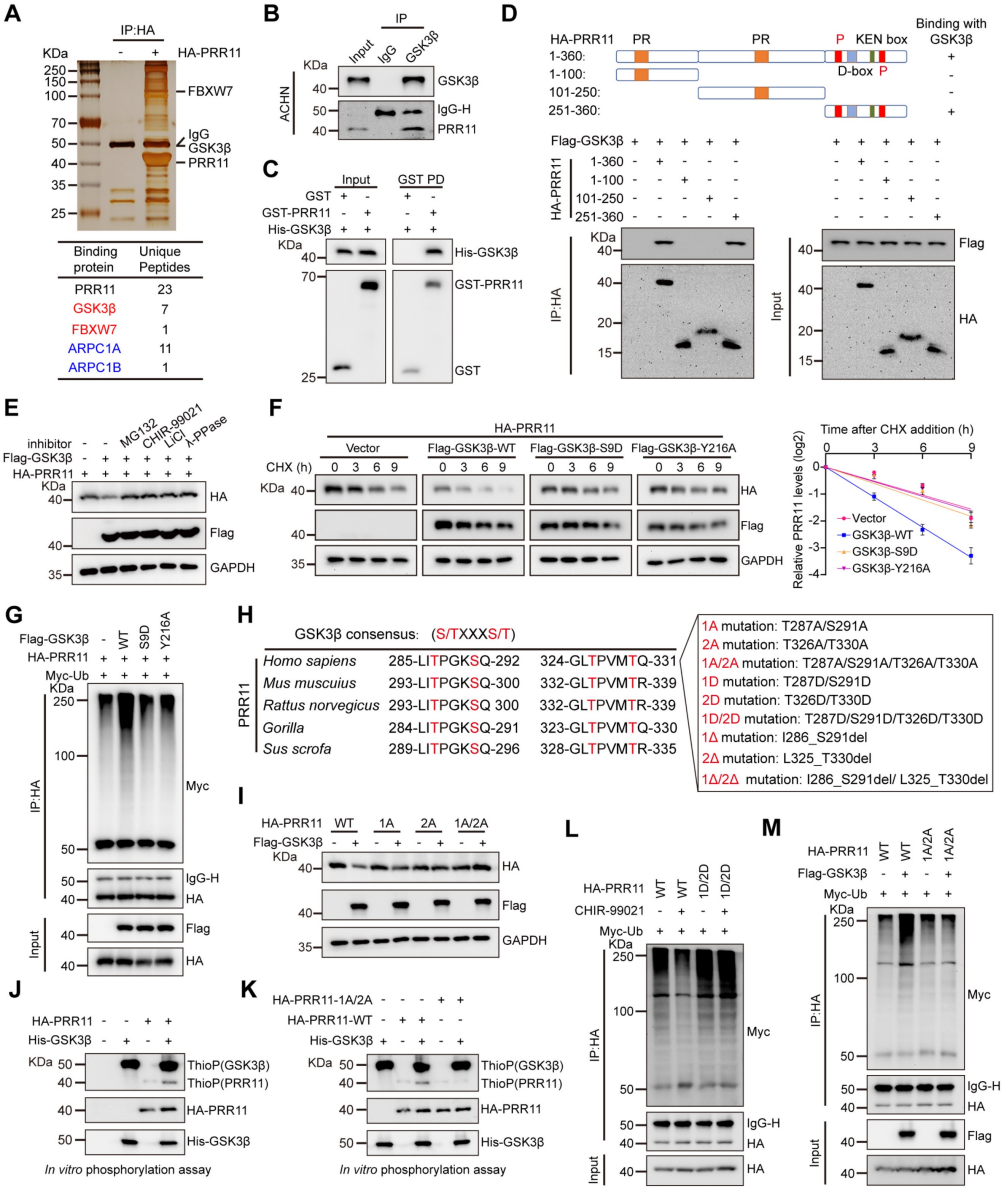
** GSK3β interacts with PRR11 to regulate PRR11 stability. (A)** HA-PRR11 plasmid was overexpressed in 293T cells, and the results were analyzed by co-IP with HA antibody, followed by silver staining (top) and mass spectrometry (bottom).** (B)** Western blot analysis of PRR11 in ACHN cells after IP analysis with GSK3β antibody. **(C)** Western blot analysis of His-GSK3β, GST-PRR11 after GST pull-down assays.** (D)** Diagram of the truncation of PRR11 (top). Full-length or truncated mutants of HA-PRR11 and Flag-GSK3β plasmid were transfected into 293T cells, and Western blot analysis of Flag-GSK3β was performed after IP analysis with HA antibody (bottom). PR: Proline-rich domain; P: CDC4 Phosphodegron (CPD) motif; D-box: D-box motif; KEN box: KEN box motif.** (E)** MG132 (10 μM), λ-PPase (8 U/μL), or GSK3β inhibitors CHIR-99021 (10 μM) and LiCl (20 mM) were added to 293T cells transfected with HA-PRR111 and Flag-GSK3β, after which Western blot analysis of HA-PRR11 was performed.** (F)** After exposing 293T cells transfected with HA-PRR11, Flag-GSK3β-WT, or Flag-GSK3β-Mut (GSK3β-S9D and GSK3β-Y216A) to CHX (50 μg/mL) for the indicated durations, Western blot analysis was conducted for HA-PRR11 (left). Quantification of PRR11 half-life (right, n = 3 biologically independent experiments).** (G)** 293T cells transfected with HA-PRR11, Myc-Ub, Vector, Flag-GSK3β-WT, or Flag-GSK3β-Mut were incubated with MG132 (10 μM) for 6 h, and Western blot analysis of Myc-Ub was performed after IP analysis with HA antibody.** (H)** GSK3β phosphorylation recognition sequence was compared with the conserved PRR11 sequence (left). Design the corresponding PRR11 dephosphorylation mimic mutants (PRR11-1A: T287A/S291A, PRR11-2A: T326A/T330A, PRR11-1A/2A: T287A/S291A/T326A/T330A), PRR11 phosphorylation mimic mutants (PRR11-1D: T287D/S291D, PRR11-2D: T326D/T330D, PRR11-1D/2D: T287D/S291D/T326D/T330D) and PRR11 phosphorylation motif deletion mutants (PRR11-1Δ: I286_S291del, PRR11-2Δ: L325_T330del, PRR11-1Δ/2Δ: I286_S291del/L325_T330del) according to the conserved sequence recognized by PRR11 (right). **(I)** 293T cells were transfected with the indicated plasmids and Western blot analysis of HA-PRR11 was performed.** (J)** HA-PRR11-WT was phosphorylated *in vitro* with active GSK3β and ATP-γ-S, and immunoblotted after alkylation with PNBM. **(K)** HA-PRR11-WT and HA-PRR11-1A/2A were phosphorylated *in vitro* with active GSK3β and ATP-γ-S, and immunoblotted after alkylation with PNBM. **(L)** 293T cells transfected with Myc-Ub, HA-PRR11-WT, or HA-PRR11-1D/2D were treated with DMSO or CHIR-990211 (10 μM) and incubated with MG132 (10 μM) for 6 h before cell collection, and then Western blot analysis of Myc-Ub was performed after IP analysis with HA antibody. **(M)** 293T cells transfected with Myc-Ub, Flag-GSK3β, HA-PRR11-WT, or HA-PRR11-1A/2A were incubated with MG132 (10 μM) for 6 h, and Western blot analysis of Myc-Ub was performed after IP analysis with HA antibody. Protein levels were quantitatively detected with ImageJ software, and linear regression was used to analyze the protein half-life (F). Data are presented as mean values ± SD.

Previously, we demonstrated that PRR11 is an independent and adverse prognostic factor for RCC that promotes RCC tumorigenesis 7. Given the potential importance of PRR11 in RCC, we aimed to identify our PRR11-binding partners of interest. HA-PRR11 was transfected into 293T cells, followed by IP-MS analysis. We identified GSK3β as a potential interacting factor for PRR11, and in addition, previously reported PRR11 interacting proteins such as ARPC1A and ARPC1B were also identified, confirming the validity of our screen for identifying PRR11 interacting proteins 13 (Figure 1A, Figure S1A and Dataset S1). Co-immunoprecipitation (co-IP) assays confirmed the interaction between exogenous HA-PRR11 and Flag-GSK3β (Figure S1B), which was further validated in RCC cells for endogenous PRR11 and GSK3β interactions (Figure 1B and Figure S1C-D). Importantly, GST pull-down assay showed direct binding of recombinant GST-PRR11 and recombinant His-GSK3β *in vitro* (Figure 1C). To explore the precise binding region of PRR11 and GSK3β more deeply, we constructed fragmented HA-PRR11 for co-IP assays and revealed that the amino acids 251-360 of PRR11 could interact with GSK3β (Figure 1D). Interestingly, this region contains numerous ubiquitination and phosphorylation sites, including two CPD motifs. Considering the reported susceptibility of PRR11 to proteasomal degradation 9 and the binding of GSK3β to CPD motif-containing regions, we hypothesized that GSK3β might mediate PRR11 degradation via phosphorylation. Subsequent investigations revealed that GSK3β overexpression attenuated the steady-state expression of PRR11, an effect effectively blocked by proteasome inhibitor MG132, λ-protein phosphatase (λ-PPase), and the GSK3β inhibitors CHIR-99021 and LiCl (Figure 1E). Moreover, knockdown or overexpression of GSK3β in ACHN and Caki-1 cells increased or decreased PRR11 protein levels, respectively, without affecting *PRR11* mRNA levels (Figure S1E-G). We then found that CHIR-99021 treatment reduced the degradation rate of endogenous PRR11 in ACHN cells (Figure S1H). Notably, GSK3β overexpression significantly promoted the degradation rate of exogenous HA-PRR11 protein, whereas the catalytic-deficient mutant of GSK3β (GSK3β-S9D and GSK3β-Y216A) failed to accelerate PRR11 degradation (Figure 1F and Figure S1I). Additionally, *in vivo* ubiquitination assays showed that GSK3β overexpression promoted PRR11 ubiquitination in a dose-dependent manner, whereas the catalytic-deficient mutant of GSK3β significantly inhibited PRR11 ubiquitination (Figure 1G and Figure S1J).

GSK3β typically binds to a substrate-specific motif (S/T-X-X-X-S/T) to catalyze phosphorylation 24. Therefore, we conducted a search and identified a GSK3β-specific phosphorylation motif in the PRR11 protein sequence that is conserved in mammals. Therefore, we constructed PRR11 dephosphorylation mimic mutants (PRR11-1A: T287A/S291A, PRR11-2A: T326A/T330A, PRR11-1A/2A: T287A/S291A/T326A/T330A), PRR11 phosphorylation mimic mutants (PRR11-1D: T287D/S291D, PRR11-2D: T326D/T330D, PRR11-1D/2D: T287D/S291D/T326D/T330D) and PRR11 CPD motif deletion mutants (PRR11-1Δ: I286_S291del, PRR11-2Δ: L325_T330del, PRR11-1Δ/2Δ: I286_S291del/L325_T330del) (Figure 1H). Notably, while GSK3β overexpression attenuated the protein levels of PRR11-WT, it did not affect the protein levels of dephosphorylation mimic mutants (Figure 1I). To investigate whether GSK3β could directly phosphorylate PRR11, we performed an* in vitro* phosphorylation assay using active GSK3β and showed that PRR11 could be directly phosphorylated by GSK3β (Figure 1J). In addition, mutating T287/S291/T326/T330 of PRR11 to alanine largely reduced the phosphorylation of PRR11 by GSK3β, indicating that T287/S291/T326/T330 of PRR11 are indeed phosphorylation sites for GSK3β (Figure 1K). Similarly, the GSK3β inhibitor CHIR-99021 inhibited the ubiquitination of PRR11-WT but not PRR11-1D/2D (Figure 1L). In addition, PRR11-WT underwent GSK3β-mediated ubiquitination, whereas PRR11-1A/2A mutants resisted the effects of GSK3β (Figure 1M). Collectively, these results suggest that GSK3β mediates the ubiquitination and degradation of PRR11 by phosphorylating the T287/S291/T326/T330 sites of PRR11.

### FBXW7 destabilizes PRR11 via ubiquitination

**Figure 2:**
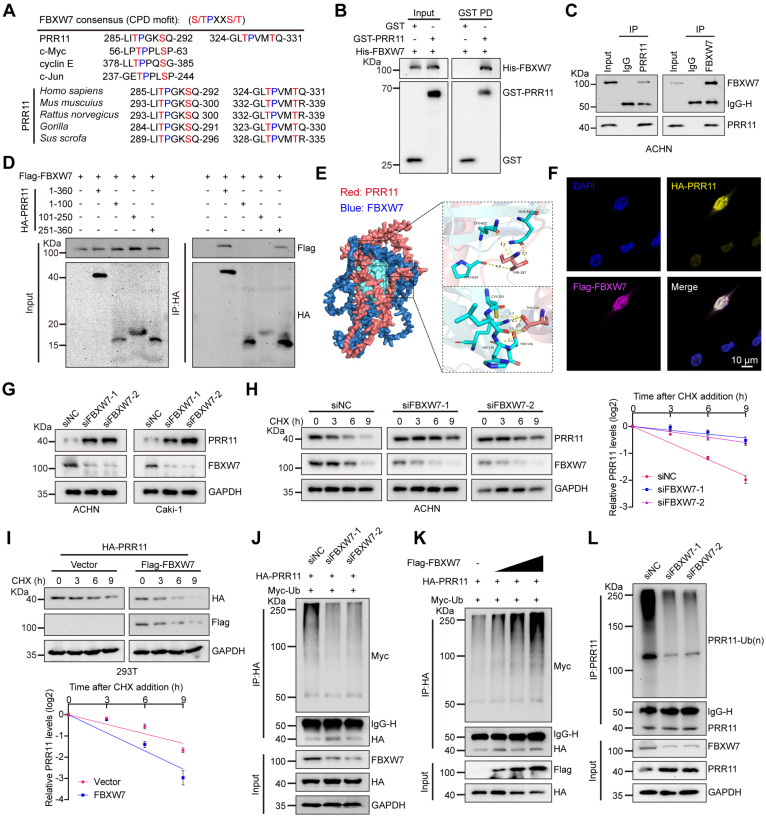
** FBXW7 promotes ubiquitination and degradation of PRR11. (A)** Comparison of conserved PRR11 sequences with known CPD motifs recognized by FBXW7. **(B)** Western blot analysis of His-FBXW7, GST-PRR11 after GST pull-down assays.** (C)** Western blot analysis of PRR11 and FBXW7 in ACHN cells after IP analysis with PRR11 (left) and FBXW7 (right) antibodies.** (D)** Full-length or truncated mutants of HA-PRR11 and Flag-FBXW7 plasmid were transfected into 293T cells, and Western blot analysis of Flag-FBXW7 was performed after IP analysis with HA antibody. **(E)** Protein docking model of FBXW7 (blue) and PRR11 (red). Hydrogen bonds are shown in yellow, and the WD40 domain of FBXW7 is shown in cyan.** (F)** Subcellular localization of overexpression of HA-PRR11 (yellow) and Flag-FBXW7 (magenta) in ACHN cells was detected by immunofluorescence.** (G)** Western blot analysis of PRR11 expression in RCC cells after *FBXW7* knockdown.** (H)** ACHN cells transfected with *siFBXW7-1* or *siFBXW7-2* were treated with CHX (50 μg/mL) for the indicated durations, after which Western blot analysis of PRR11 expression was performed (left). PRR11 half-life quantification (right, n = 3 biological independent experiments).** (I)** 293T cells transfected with Flag-FBXW7 and HA-PRR11 were treated with CHX (50 μg/mL) for indicated durations, after which Western blot analysis was performed for HA-PRR11 (top). Quantification of PRR11 half-life (bottom, n = 3 biologically independent experiments).** (J)** 293T cells transfected with *siFBXW7*, HA-PRR11, and Myc-Ub were incubated with MG132 (10 μM) for 6 h, and Western blot analysis of Myc-Ub was performed after IP analysis with HA antibody.** (K)** 293T cells transfected with Flag-FBXW7 (0.2 μg, 1.0 μg, or 4.0 μg), HA-PRR11, and Myc-Ub were incubated with MG132 (10 μM) for 6 h, and Western blot analysis of Myc-Ub was performed after IP analysis with HA antibody. **(L)** *FBXW7* knockdown ACHN cells were incubated with MG132 (10 μM) for 6 h, IP analysis using PRR11 antibody and Western blot analysis to detect endogenous ubiquitination. Protein levels were quantitatively detected with ImageJ software, and Linear Regression for analyzing protein half-life (H-I). Data are presented as mean values ± SD.

Given the role of FBXW7 in degrading various key proteins, such as c-MYC, CCNE1, and c-JUN, through recognition of their CPD (T/S-P-X-X-S/T) motifs and since the phosphorylation site of GSK3β on PRR11 coincides with the FBXW7-binding motif (Figure 2A), we explored FBXW7 as a potential interacting protein of PRR11 through IP-MS analysis (Figure 1A and Figure S2A). Therefore, we identified FBXW7 as a potential E3 ligase involved in GSK3β-mediated PRR11 degradation. Indeed, we demonstrated a direct binding relationship between recombinant GST-PRR11 and recombinant His-FBXW7 by GST pull-down assay (Figure 2B). Next, we confirmed the interaction of FBXW7 with PRR11 using both endogenous and exogenous co-IP assays (Figure 2C and Figure S2B-C). Additionally, similar to GSK3β, FBXW7 binds to PRR11 via its amino acid domain 251-360 (Figure 2D). Protein molecular docking simulation experiments also showed that the C-terminal structural domain of PRR11 mainly interacts with the WD40 structural domain of FBXW7. Fine-structure interaction data indicated that Thr287 and Thr326 of PRR11 interact tightly with the WD40 structural domain of FBXW7 through multiple hydrogen bonds (Figure 2E). Table S1 showed the detailed information of the molecular docking interaction site. Immunofluorescence analysis revealed that FBXW7 and PRR11 colocalized in the nucleus (Figure 2F). Silencing *FBXW7* in ACHN and Caki-1 cells resulted in elevated PRR11 protein expression, whereas overexpression of FBXW7 led to decreased PRR11 protein levels (Figure 2G and Figure S2D), with no effect on *PRR11* mRNA levels (Figure S2E). Furthermore, PRR11 protein levels were reduced by FBXW7 in a dose-dependent manner (Figure S2F), and proteasome inhibitor MG132 prevented FBXW7 from degrading the PRR11 protein (Figure S2G). Consistently, silencing *FBXW7* in RCC cells slowed the PRR11 half-life (Figure 2H and Figure S2H), whereas overexpressing FBXW7 in 293T cells accelerated the PRR11 half-life (Figure 2I). Moreover, silencing *FBXW7* attenuated PRR11 ubiquitination in 293T cells (Figure 2J), whereas overexpression of FBXW7 promoted PRR11 ubiquitination in a dose-dependent manner (Figure 2K). Similarly, *FBXW7* silencing in ACHN cells reduced endogenous PRR11 ubiquitination (Figure 2L). These findings collectively indicate that FBXW7 targets PRR11 for ubiquitination and subsequent degradation.

### FBXW7-mediated destabilization of PRR11 via its CPD motif

**Figure 3:**
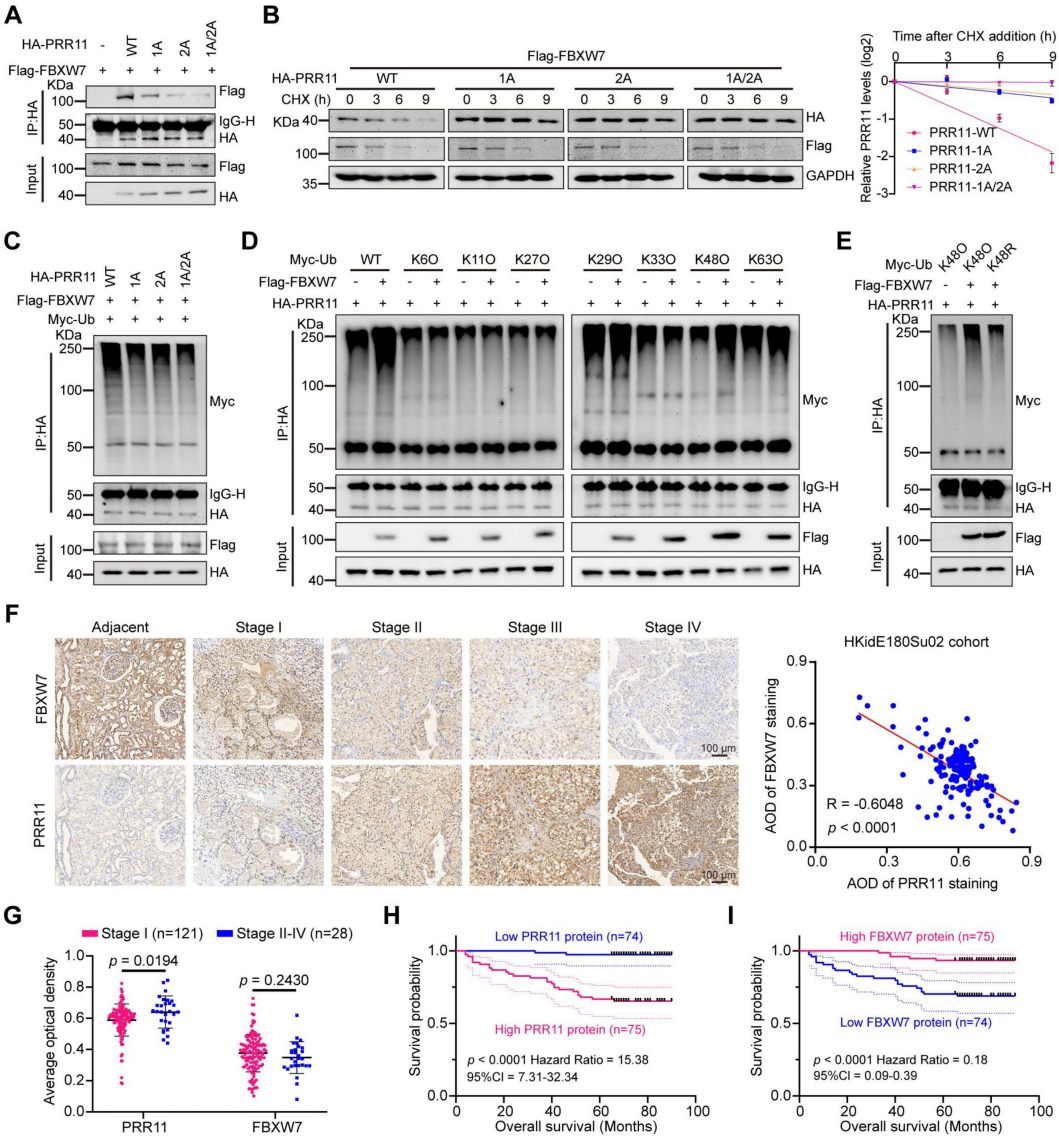
** FBXW7 degrades PRR11 via the CPD motif of PRR11. (A)** Flag-FBXW7, Vector, HA-PRR11-WT, or PRR11 dephosphorylation mimic mutants (HA-PRR11-1A, HA-PRR11-2A, and HA-PRR11-1A/2A) plasmid were transfected into 293T cells. After IP analysis with HA antibody, Western blot analysis of Flag-FBXW7 was performed.** (B)** 293T cells transfected with Flag-FBXW7, HA-PRR11-WT, or PRR11 dephosphorylation mimic mutants were treated with CHX (50 μg/mL) for indicated times, and then Western blot analysis was performed for HA-PRR11 (left). Quantification of PRR11 half-life (right, n = 3 biologically independent experiments). **(C)** 293T cells transfected with Flag-FBXW7, Myc-Ub, HA-PRR11-WT, or PRR11 dephosphorylation mimic mutants were incubated with MG132 (10 μM) for 6 h, and Western blot analysis of Myc-Ub was performed after IP analysis with HA antibody.** (D)** 293T cells transfected with HA-PRR11, Vector, Flag-FBXW7, Myc-Ub-WT, or Myc-Ub-Mut (K6O, K11O, K27O, K29O, K33O, K48O, or K63O) were incubated with MG132 (10 μM) for 6 h, and Western blot analysis of Myc-Ub was performed after IP analysis with HA antibody. K6O, K11O, K27O, K29O, K33O, K48O, and K63O appearing represent ubiquitin mutants (including K6-only, K11-only, K27-only, K29-only, K33-only, K48-only, and K63-only) that retain only a single lysine residue, respectively.** (E)** 293T cells transfected with HA-PRR11, Myc-Ub-Mut (K48O or K48R), Vector, or Flag-FBXW7 were incubated with MG132 (10 μM) for 6 h, and Western blot analysis of Myc-Ub was performed after IP analysis with HA antibody. K48O and K48R appearing represent ubiquitin mutant retaining only the K48 lysine residue and ubiquitin mutant in which only lysine residue 48 is mutated to arginine, respectively.** (F)** Representative images of immunohistochemical staining of PRR11 and FBXW7 proteins in HKidE180Su02 cohort in RCC adjacent tissue and each AJCC stage (The seventh edition of AJCC: stage I, II, III, IV) (left). 149 RCC samples were quantified and the correlation between PRR11 and FBXW7 protein levels was analyzed based on Pearson test (right). **(G)** Statistical plots of the expression levels of PRR11 and FBXW7 in HKidE180Su02 cohort in different AJCC stages. **(H-I)** The RCC samples were divided into high expression and low expression groups based on median PRR11 and FBXW7 protein levels, respectively, and survival analyses were performed by the log-rank test of Kaplan-Meier analysis. PRR11 average optical density (AOD) = integral optical density (IOD)/area. Protein levels were quantitatively detected with ImageJ software, and linear regression was used to analyze the protein half-life (B). The *p*-values were calculated with two-tailed Student's t-test (G). Data are presented as mean values ± SD.

By further investigating whether the CPD motif of PRR11 is associated with FBXW7 binding and ubiquitination, we found that PRR11 dephosphorylation mimic mutants and CPD motif deletion mutants reduced the interaction of FBXW7 with PRR11 (Figure 3A and Figure S3A), whereas PRR11 phosphorylation mimic mutants enhanced this interaction (Figure S3B). Importantly, PRR11 dephosphorylation mimic mutants and CPD motif deletion mutants presented a prolonged protein half-life and inhibited ubiquitination (Figure 3B-C and Figure S3C, E), whereas phosphorylation mimic mutants presented a shorter half-life and enhanced ubiquitination (Figure S3D, F).

FBXW7 is frequently mutated in human cancers, with R505 and R479 identified as common mutation hotspots 16. To investigate their effects, we constructed FBXW7-R505C and FBXW7-R479Q mutant plasmids. Additionally, an FBXW7 catalytic-deficient mutant (FBXW7-ΔF-box) was constructed to examine the role of FBXW7 catalytic activity in regulating PRR11. Compared to FBXW7-WT, both FBXW7-R505C and FBXW7-R479Q mutants exhibited significantly reduced binding to PRR11 and inhibited the degradation of PRR11. Although FBXW7-ΔF-box retained its binding ability to PRR11, it significantly weakened the degradation of PRR11 (Figure S4A-B). Furthermore, all FBXW7 mutants reduced the ubiquitination of PRR11 and extended its protein half-life (Figure S4C-D). To determine the type of ubiquitin chain in which PRR11 is ubiquitinated by FBXW7, we generated a series of mutant ubiquitin plasmids. The results showed that FBXW7 efficiently catalyzed the K48-linked ubiquitin chain on PRR11 (Figure 3D), whereas the K48R ubiquitin mutant blocked FBXW7-induced ubiquitination of PRR11 (Figure 3E). These findings collectively suggest that phosphorylation of the PRR11 CPD motif is essential for FBXW7-mediated K48 ubiquitination of PRR11.

To further characterize the relationship between PRR11 and FBXW7, we quantified PRR11 and FBXW7 protein expression by IHC in 149 clinical RCC samples from the HKidE180Su02 cohort and found that there was a significant negative correlation between PRR11 and FBXW7 protein levels (Figure 3F). In addition, PRR11 expression was significantly positively correlated with tumor stage and pathological grade, and although FBXW7 expression did not significantly differ with tumor stage or tumor size, it exhibited a negative correlation trend (Figure 3G and Table S2-S3). In terms of prognosis, PRR11 expression was strongly correlated with the poor prognosis of RCC patients, and in contrast to PRR11, high expression of FBXW7 significantly improved the overall survival of RCC patients (Figure 3H-I). These findings indicate that PRR11 expression is negatively correlated with FBXW7 expression in RCC and that higher PRR11 expression is associated with advanced tumor stage, pathological grade, and poor prognosis, whereas elevated FBXW7 expression improves overall survival in RCC patients.

### FBXW7 promotes PRR11 ubiquitination in a GSK3β-mediated phosphorylation-dependent manner

**Figure 4:**
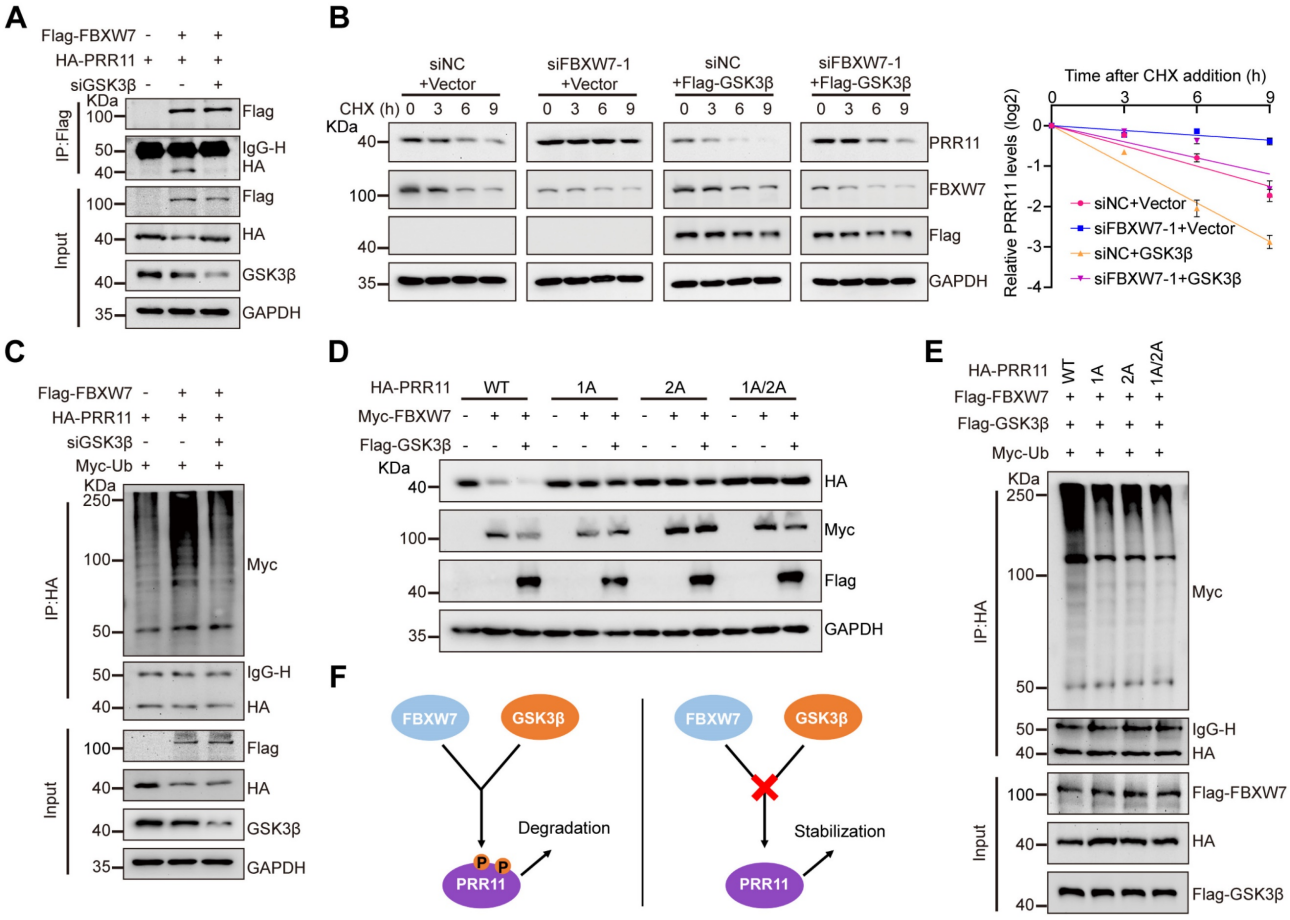
** FBXW7 promotes ubiquitination and degradation of PRR11 via GSK3β-mediated phosphorylation. (A)** HA-PRR11, Flag-FBXW7, or Flag-FBXW7+*siGSK3β* were transfected into 293T cells. After IP analysis with Flag antibody, Western blot analysis was performed on HA-PRR11.** (B)** 293T cells transfected with *siFBXW7-1*, Flag-GSK3β, or *siFBXW7-1+*Flag-GSK3β were incubated with CHX (50 μg/mL) for indicated times, and then Western blot analysis was performed for PRR11 (left). Quantification of PRR11 half-life (right, n = 3 biologically independent experiments).** (C)** 293T cells transfected with HA-PRR11, Myc-Ub, Flag-FBXW7, or Flag-FBXW7+*siGSK3β* were treated with MG132 (10 μM) for 6 h, and Western blot analysis of Myc-Ub was performed after IP analysis with HA antibody. **(D)** 293T cells were transfected with Myc-FBXW7, Flag-GSK3β, HA-PRR11-WT, or PRR11 dephosphorylation mimic mutants (HA-PRR11-1A, HA-PRR11-2A, and HA-PRR11-1A/2A), and Western blot analysis of PRR11 was performed.** (E)** 293T cells transfected with Myc-Ub, Flag-FBXW7, Flag-GSK3β, HA-PRR11-WT, or PRR11 dephosphorylation mimic mutants were treated with MG132 (10 μM) for 6 h, and Western blot analysis of Myc-Ub was performed after IP analysis with HA antibody. **(F)** Schematic illustration of FBXW7-GSK3β can only promote phosphorylated PRR11 degradation. Protein levels were quantitatively detected with ImageJ software, and linear regression was used to analyze the protein half-life (B).

Subsequently, we continued to explore the synergistic regulation of PRR11 by GSK3β and FBXW7. By GST pull-down assay, we found that there is no competition for binding of GSK3β and FBXW7 to PRR11 interactions (Figure S5A). Interestingly, the interaction between FBXW7 and PRR11 was attenuated by the addition of λ-PPase to cellular protein extracts (Figure S5B). More importantly, both CHIR-99021 treatment and *GSK3β* knockdown weakened the interactions between FBXW7 and PRR11 (Figure 4A and Figure S5C). Conversely, overexpression of GSK3β enhanced the FBXW7-PRR11 interaction (Figure S5D). As expected, in *FBXW7*-downregulated cells, GSK3β overexpression rescued the effect of *FBXW7* silencing on the PRR11 protein level (Figure 4B). Consistently, CHIR-99021 treatment significantly prevented the decrease in PRR11 abundance induced by FBXW7 overexpression (Figure S5E). Furthermore, knockdown of *GSK3β* or treatment with CHIR-99021 abolished FBXW7-mediated PRR11 ubiquitination (Figure 4C and Figure S5F), whereas GSK3β overexpression enhanced FBXW7-mediated PRR11 ubiquitination (Figure S5G).

Notably, PRR11 dephosphorylation mimic mutants abrogated the synergistic degradation of PRR11 protein levels by FBXW7 and GSK3β (Figure 4D). In addition, these mutants attenuated the degree of PRR11 ubiquitination mediated by the co-expression of FBXW7 and GSK3β compared to PRR11-WT (Figure 4E). Similarly, the GSK3β/FBXW7-mediated ubiquitination of PRR11 at K48 was inhibited by these mutants (Figure S5H). This further demonstrated the necessity of PRR11 phosphorylation for FBXW7-mediated degradation (Figure 4F). In summary, our results suggest that FBXW7 collaborates with GSK3β to promote PRR11 ubiquitination and degradation.

### *PRR11* silencing induced oxidative DNA damage in RCC cells

**Figure 5:**
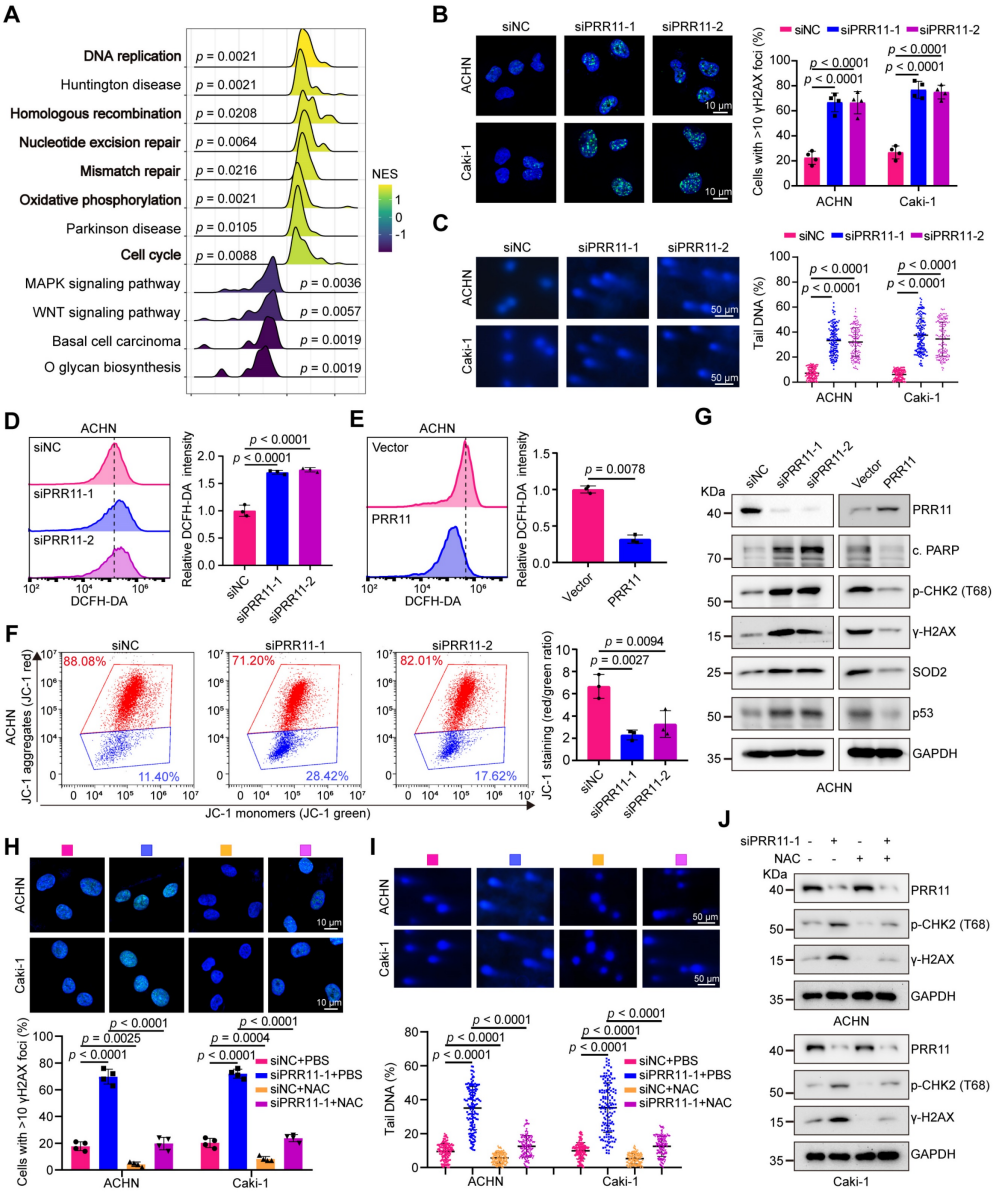
***PRR11* knockdown promotes oxidative DNA damage in RCC. (A)** RNA-seq analysis was performed on *PRR11* knockdown ACHN cells, followed by GSEA for all genes. GSEA was performed using the R package “clusterProfiler”. Statistical significance was determined by two-tailed Fisher's exact test, and the *p*-value was corrected by Benjamini & Hochberg's method. The top 12 gene sets were selected according to p.adjust and the normalized enrichment score (NES) is displayed. **(B)** γH2AX staining after *PRR11* silencing in ACHN and Caki-1 cells was analyzed by immunofluorescence (left), and the number of γH2AX foci was quantified (right, n = 4 biologically independent experiments). **(C)** Representative images of alkaline comet assays after *PRR11* silencing in ACHN and Caki-1 cells (left). ACHN and Caki-1 cells per group (n = 150) were quantified using CASP software (right, n = 3 biologically independent experiments).** (D-E)** ROS levels in ACHN cells after *PRR11* knockdown or overexpression were detected using flow cytometry (left), followed by statistical analysis (right, n = 3 biologically independent experiments). **(F)** Flow cytometry detection of MMP levels in ACHN cells after *PRR11* silencing (left), followed by statistical analysis (right, n = 3 biologically independent experiments).** (G)** Western blot analysis of DNA damage markers and oxidative stress markers after *PRR11* silencing or PRR11 overexpression in ACHN cells. **(H)** γH2AX staining after *PRR11* silencing or/and ROS scavenger NAC (5 mM) treatment in ACHN and Caki-1 cells was analyzed by immunofluorescence (top), and the number of γH2AX foci was quantified (bottom, n = 4 biologically independent experiments).** (I)** Representative images of alkaline comet assays after *PRR11* silencing or/and ROS scavenger NAC (5 mM) treatment in ACHN and Caki-1 cells (top). ACHN and Caki-1 cells per group (n = 150) were quantified using CASP software (bottom, n = 3 biologically independent experiments).** (J)** Western blot analysis of DNA damage markers after *PRR11* silencing or/and ROS scavenger NAC (5 mM) treatment in ACHN and Caki-1 cells. The *p*-values were calculated with one-way ANOVA with Dunnett's multiple comparisons test (B-D, F), one-way ANOVA with Tukey's multiple comparisons test (H-I) and two-tailed Student's t-test (E). Data are presented as mean values ± SD.

Numerous studies have shown that PRR11 is involved in cell cycle arrest and apoptotic processes 7, 8, 25. Notably, stress-induced DNA damage can trigger both apoptosis and cell cycle arrest 26. Through Pearson correlation analysis of TCGA-KIRC data, we identified the top 200 genes closely related to PRR11 (Dataset S2). Subsequent functional annotation analyses (GO and KEGG) revealed enrichment of PRR11 in processes related to homologous recombination, DNA replication, DNA double-strand breaks, DNA damage and cell cycle (Figure S6A-B, Table S4-S5 and Dataset S3). RNA-seq assays of *PRR11*-silenced ACHN cells corroborated these findings and, as indicated by GSEA, revealed associations between PRR11 and DNA replication/damage/repair, oxidative phosphorylation, and cell cycle (Figure 5A and Table S6 and Dataset S4).

To further investigate the role of PRR11 in RCC, we established and validated knockdown and overexpression RCC cell lines (Figure S6C-D). Consistent with these findings, the knockdown of *PRR11* increased the number of foci with the DNA damage marker γH2AX, whereas the overexpression of PRR11 reduced the number of foci with this marker (Figure 5B and Figure S6E). Direct observation of DNA damage via the alkaline comet assay confirmed that *PRR11* silencing increased the comet tail DNA content, whereas PRR11 overexpression decreased the comet tail moment (Figure 5C and Figure S6F). Given the pivotal role of ROS in mitochondria-mediated apoptosis and DNA damage 27, we investigated the impact of PRR11 on ROS levels in RCC cells. Flow cytometry analysis revealed that *PRR11* knockdown increased ROS levels, whereas PRR11 overexpression inhibited ROS production (Figure 5D-E and Figure S7A-B). Moreover, assessment of the mitochondrial membrane potential (MMP) using JC-1 revealed fewer JC-1 aggregates in *PRR11* knockdown RCC cells and more JC-1 aggregates in PRR11 overexpressing RCC cells, suggesting a reduced MMP upon* PRR11* silencing (Figure 5F and Figure S7C-E). Western blot analysis further supported these findings, showing increased levels of cleaved PARP, pT68-CHK2, γ-H2AX, SOD2 and p53 in *PRR11*-silenced RCC cells, whereas PRR11 overexpression had the opposite effect (Figure 5G and Figure S7F).

Excessive accumulation of ROS often triggers DNA damage in cells 28. To investigate whether *PRR11* knockdown-induced DNA damage is caused by elevated ROS, we treated RCC cells with the ROS scavenger NAC. NAC treatment significantly inhibited ROS accumulation caused by* PRR11* knockdown (Figure S8A-B). Importantly, the addition of NAC counteracted the *PRR11* knockdown-induced decrease in MMP and increase in DNA damage (Figure 5H-J and Figure S8C-D). Thus, these data collectively suggest that *PRR11* knockdown promotes DNA damage through ROS accumulation in RCC cells.

### PRR11 regulates the AKT pathway and activated AKT stabilizes PRR11 protein

**Figure 6:**
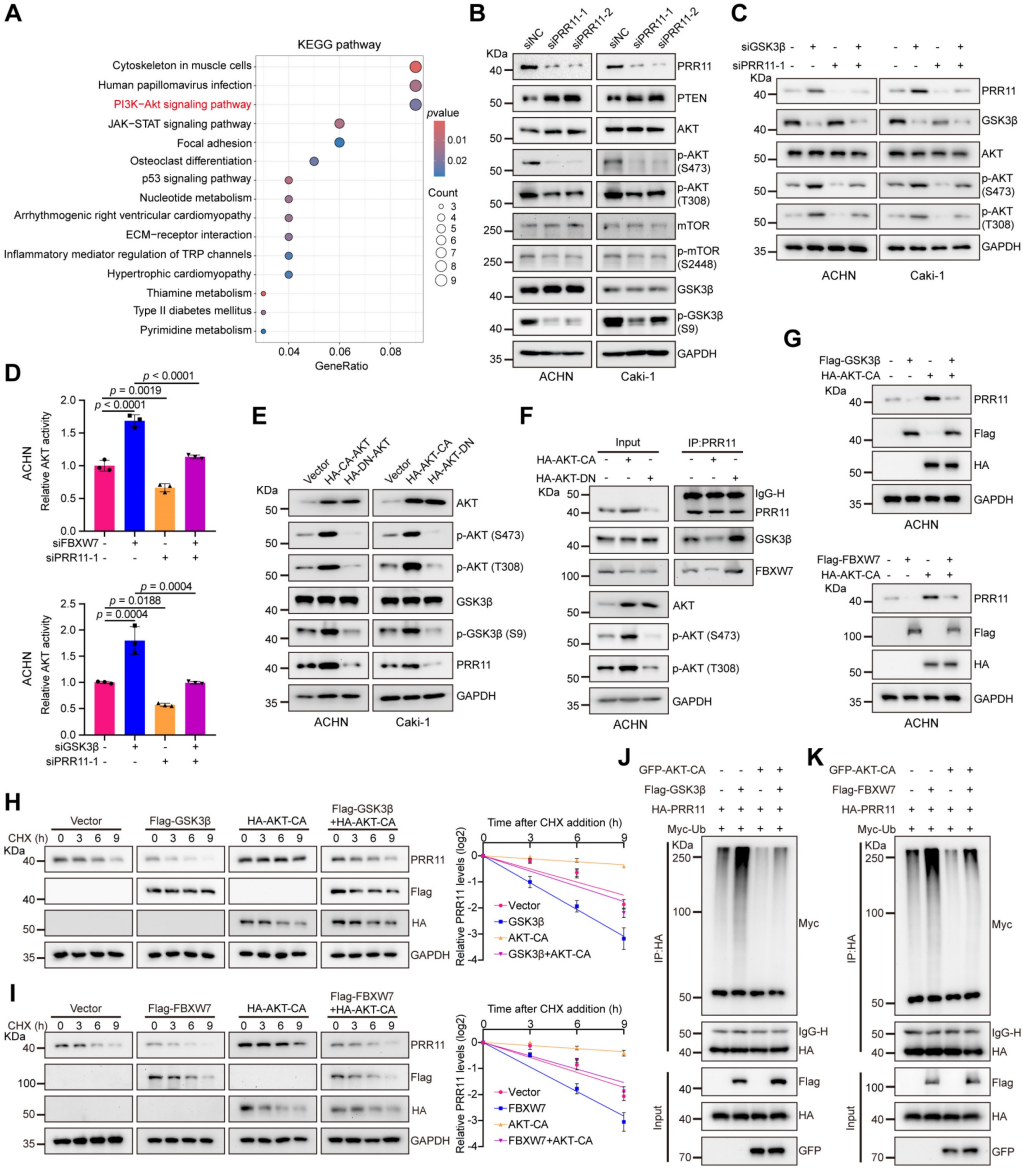
** FBXW7/GSK3β-PRR11 axis activates the AKT pathway and AKT activation inhibits PRR11 degradation. (A)** KEGG pathway enrichment analysis was performed on DEGs in the RNA-seq expression matrix of *PRR11* silencing ACHN cells. KEGG analysis was performed using the R package "clusterProfiler". The *p*-value was calculated by two-tailed Fisher's exact test, and *p* < 0.05 was used as a screening criterion.** (B)** Protein levels of each important factor of the AKT signaling pathway were analyzed by Western blot analysis after *PRR11* knockdown in RCC cells. **(C)** Western blot analysis of AKT phosphorylation levels after transfection of ACHN and Caki-1 cells with *siGSK3β* or/and *siPRR11*.** (D)** AKT activity was measured in ACHN cells transfected with* siFBXW7* (top)*/siGSK3β* (bottom) or/and *siPRR11* (n = 3 biologically independent experiments).** (E)** Changes in PRR11 protein levels were analyzed by Western blot analysis after transfection of AKT-CA or AKT-DN in RCC cells.** (F)** AKT-CA or AKT-DN plasmid was transfected into ACHN cells. After IP analysis with PRR11 antibody, Western blot analysis was performed on GSK3β and FBXW7.** (G)** Western blot analysis of PRR11 after transfection of ACHN cells with Flag-GSK3β (top)/Flag-FBXW7 (bottom) or/and HA-AKT-CA.** (H)** 293T cells transfected with the Flag-GSK3β or/and HA-AKT-CA were treated with CHX (50 μg/mL) for the indicated durations, after which Western blot analysis was performed for PRR11 (left). Quantification of the PRR11 half-life (right, n = 3 biologically independent experiments).** (I)** 293T cells transfected with the Flag-FBXW7 or/and HA-AKT-CA were treated with CHX (50 μg/mL) for the indicated durations, after which Western blot analysis was performed for PRR11 (left). Quantification of the PRR11 half-life (right, n = 3 biologically independent experiments).** (J)** 293T cells transfected with HA-PRR11, Myc-Ub, Flag-GSK3β, or/and GFP-AKT-CA were incubated with MG132 (10 μM) for 6 h, and Western blot analysis of Myc-Ub was performed after IP analysis with HA antibody.** (K)** 293T cells transfected with HA-PRR11, Myc-Ub, Flag-FBXW7, or/and GFP-AKT-CA were incubated with MG132 (10 μM) for 6 h, and Western blot analysis of Myc-Ub was performed after IP analysis with HA antibody. Protein levels were quantitatively detected with ImageJ software, and linear regression was used to analyze the protein half-life (H-I). The *p*-values were calculated with one-way ANOVA with Tukey's multiple comparisons test (D). Data are presented as mean values ± SD.

To explore the downstream signals of PRR11 in RCC, we further analyzed the RNA-seq data of ACHN cells after *PRR11* knockdown. KEGG pathway analysis results revealed that PRR11 was enriched in AKT signaling pathway (Figure 6A, Table S7 and Dataset S5). As expected, Western blot analysis suggested that *PRR11* silencing upregulated PTEN expression and reduced the levels of pT308-AKT and pS473-AKT, as well as those of the downstream effectors of the AKT pathway, pS2448-mTOR and pS9-GSK3β (Figure 6B). Conversely, overexpression of PRR11 had the opposite effect (Figure S9A).

Deletion of *FBXW7* synergizes with activated AKT signaling to promote cholangiocarcinogenesis. Interestingly, FBXW7 decreased the p-AKT level in some cholangiocarcinoma cell lines, whereas the dominant-negative form of FBXW7 (FBXW7-ΔF-box) significantly upregulated the p-AKT level 29. It has also been shown that in pancreatic cancer FBXW7 could interfere with AKT signaling activity by targeting SIK2 degradation 30. In addition, GSK3β-mediated phosphorylation of Rictor significantly inhibited AKT signaling was also demonstrated 31. Based on the above studies and our conclusion that FBXW7/GSK3β regulates PRR11 protein stability, we explored whether FBXW7/GSK3β has an effect on PRR11-mediated AKT activity in RCC. We found that knockdown of *FBXW7* or *GSK3β* enhanced the phosphorylation levels of AKT, an effect that could be reversed by downregulation of *PRR11* (Figure 6C and Figure S9B). Importantly, quantification of AKT activity using the AKT kinase activity kit also demonstrated that downregulation of *FBXW7* or *GSK3β* significantly enhanced AKT activity, an effect that was counteracted by *PRR11* knockdown (Figure 6D and Figure S9C).

Notably, AKT is known to phosphorylate GSK3β, resulting in its inactivation 32. Combined with previous findings showing that GSK3β promotes PRR11 degradation, we hypothesized that an AKT-GSK3β-PRR11 feedback loop is formed. To test whether AKT activation or inhibition affects the protein stability of PRR11, we transfected constitutively active AKT (AKT-CA) and dominant-negative AKT (AKT-DN) plasmids into RCC cells. The activation of AKT indeed increased the PRR11 protein level, whereas AKT inactivation attenuated the PRR11 protein level (Figure 6E). Importantly, an endogenous co-IP assay in RCC cells revealed that AKT activation attenuated PRR11 interactions with FBXW7 and GSK3β, whereas AKT inactivation strengthened these interactions (Figure 6F and Figure S9D). Then to explored whether AKT activation affects FBXW7/GSK3β-mediated ubiquitination and degradation of PRR11. We first demonstrated that activation of AKT prevented FBXW7/GSK3β-mediated PRR11 degradation by using AKT-CA in ACHN cells (Figure 6G). The in-depth mechanism of AKT on PRR11 via FBXW7/GSK3β was then continued to be explored in 293T cells. By CHX and ubiquitination assays we further found that AKT-CA inhibited the ubiquitination and degradation of PRR11 by FBXW7/GSK3β (Figure 6H-K and Figure S9E). In summary, PRR11 participates in AKT pathway activation, and activated AKT stabilizes the PRR11 protein by inactivating GSK3β, thus forming a positive feedback loop.

### PRR11-AKT axis regulates oxidative DNA damage and promotes RCC

**Figure 7:**
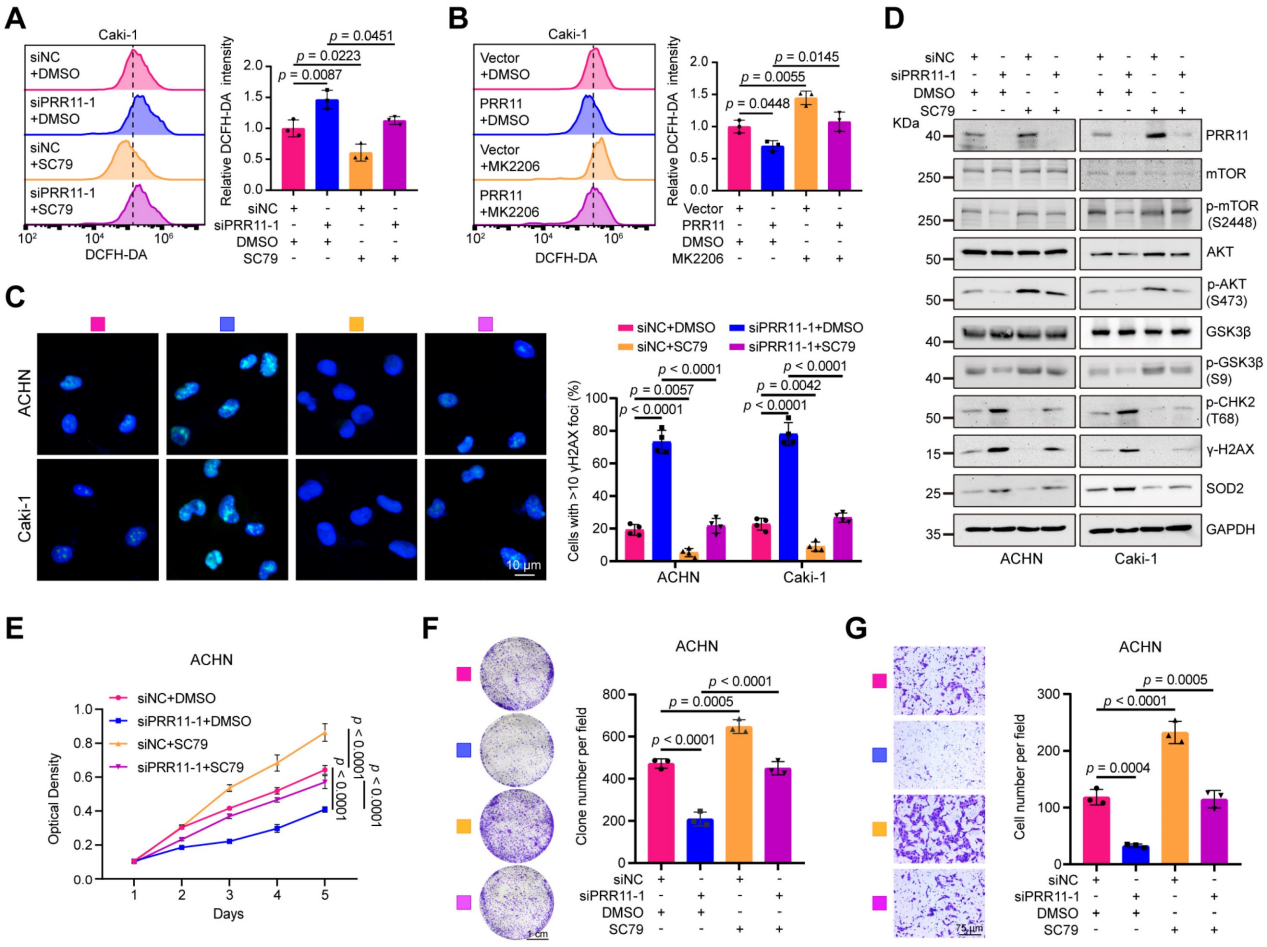
** PRR11-AKT axis regulates oxidative DNA damage and affects proliferation and migration in RCC. (A-B)** Flow cytometry detection of ROS levels from the indicated groups with *PRR11* silencing/overexpression and AKT agonist SC79 (20 μM)/AKT inhibitor MK2206 (10 μM) treatment in Caki-1 cells (left), followed by statistical analysis (right, n = 3 biologically independent experiments).** (C)** Immunofluorescence analysis of the number of γH2AX foci in RCC cells treated with or without *PRR11* silencing and/or SC79 treatment (left), followed by statistical analysis (right, n = 4 biological independent experiments).** (D)** Western blot analysis of oxidative DNA damage and AKT pathway associated protein levels in RCC cells treated with or without *PRR11* silencing and/or SC79 treatment.** (E)** MTT assay of the proliferative capacity of ACHN cells treated with or without *PRR11* silencing and/or AKT agonist SC79 treatment (n = 6 biologically independent experiments).** (F)** Colony formation assay of the proliferative capacity of ACHN cells treated with or without *PRR11* silencing and/or AKT agonist SC79 treatment (left), followed by statistical analysis (right, n = 3 biologically independent experiments).** (G)** Transwell assay showing the migration capacity of ACHN cells treated with or without *PRR11* silencing and/or AKT agonist SC79 treatment (left), followed by statistical analysis (right, n = 3 biologically independent experiments). The *p*-values were calculated with one-way ANOVA with Tukey's multiple comparisons test (A-C, E-G). Data are presented as mean values ± SD.

Subsequently, we further investigated the biological role of the PRR11-AKT axis in the tumor behavior of RCC. The addition of the AKT agonist SC79 to ACHN and Caki-1 cells rescued the increase in ROS levels in *PRR11* knockdown cells (Figure 7A and Figure S10A). Similarly, the AKT inhibitor MK2206 counteracted the decrease in ROS levels caused by PRR11 overexpression (Figure 7B and Figure S10B). Additionally, knockdown of *PRR11* increased the number of nuclear γH2AX foci, which was reversed by SC79 treatment (Figure 7C), while MK2206 treatment had the opposite effect (Figure S10C). Western blot analysis further verified that SC79 treatment rescued the upregulation of DNA oxidative damage markers (pT68-CHK2, γ-H2AX, and SOD2) and the decrease in key factors of AKT signaling pathway (pS473-AKT, pS2448-mTOR, and pS9-GSK3β) caused by *PRR11* silencing (Figure 7D). Similarly, MK2206 treatment also counteracted the effect of PRR11 overexpression on the corresponding protein (Figure S10D). This observation suggested that PRR11 regulates oxidative DNA damage in RCC by activating the AKT pathway.

To investigate whether the PRR11-AKT axis affects RCC proliferation and metastasis, we conducted colony formation and MTT assays. Silencing and overexpression of PRR11 attenuated and enhanced the proliferative capacity of ACHN and Caki-1 cells, respectively, and these effects were reversed by SC79 and MK2206 (Figure 7E-F and Figure S11A-F). In addition, SC79 treatment counteracted the decrease in migratory capacity induced by *PRR11* silencing, whereas MK2206 treatment inhibited the enhancing effect of PRR11 on migratory capacity (Figure 7G and Figure S11G-I).

**Figure 8:**
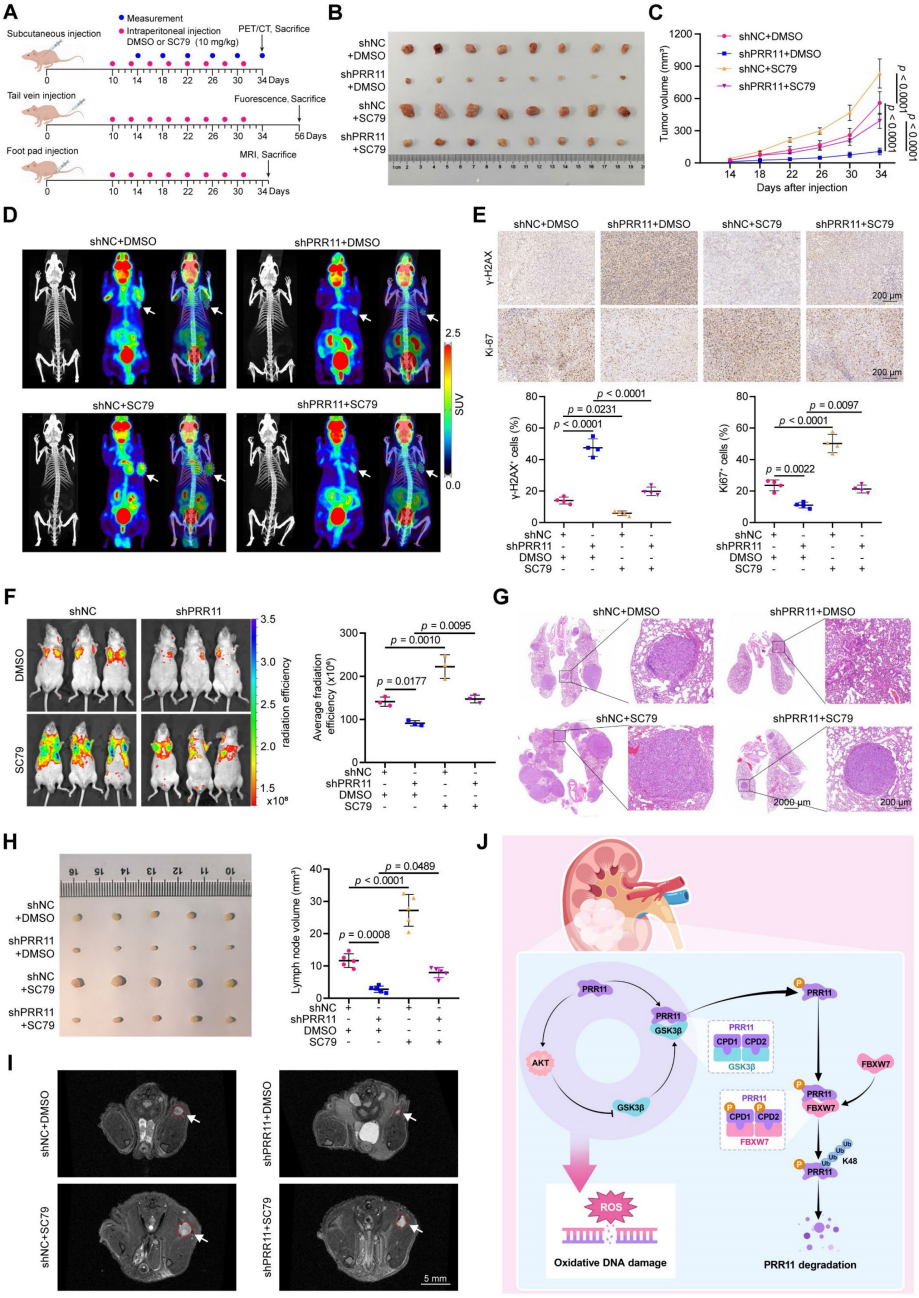
** PRR11-AKT axis promotes RCC proliferation and metastasis *in vivo*. (A)** A schematic diagram on the construction of* in vivo* models of tumor proliferation and metastasis and drug therapy. **(B)** Gross diagram of xenograft model from mice injected ACHN cells transfected with *shNC* or *shPRR11* with or without SC79 treatment.** (C)** Tumor volume statistic for mice injected subcutaneously with ACHN cells transfected with *shNC* or *shPRR11* and treated with or without SC79 (n = 8 per group).** (D)** ^18^F-FDG PET/CT scans were used to assess tumor growth and malignancy in each group of mice. Each group of images is represented from left to right as follows: CT image, PET image, PET/CT fusion image.** (E)** Representative images of Ki-67 and γ-H2AX IHC staining of tumor tissue from each group of mice in the xenograft model (top), and their quantitative statistical plots (bottom, n = 4 per group).** (F)** GFP fluorescence intensity between groups (control vs. *shPRR11* vs. SC79 vs. *shPRR11*+SC79) in the model of tail vein injection lung metastasis established using ACHN cells (left). Average radiation efficiency in lung is evaluated by ImageJ software, and statistics are performed (right, n = 3 per group).** (G)** Representative images of H&E staining of mice lung tissues from each group.** (H)** Gross diagram of a popliteal lymph node metastasis model of mice injected with ACHN cells transfected with *shNC* or *shPRR11* with or without SC79 treatment (left). Popliteal lymph node volume statistics for mice injected with ACHN cells transfected with *shNC* or *shPRR11* and treated with or without SC79 (right, n = 5 per group). **(I)** MRI T2 axial scanning imaging of popliteal lymph nodes in mice.** (J)** Mechanistic diagram of this study. FBXW7-GSK3β mediates PRR11 degradation, thereby regulating the AKT pathway. In turn, AKT-GSK3β is involved in regulating PRR11 degradation, forming a positive feedback loop that regulates oxidative DNA damage and accelerates RCC progression. The *p*-values were calculated with one-way ANOVA with Tukey's multiple comparisons test (C, E-F, H). Data are presented as mean values ± SD.

To further investigate the impact of the PRR11-AKT axis on RCC tumor behavior *in vivo*, we established *shNC* and *shPRR11* stable ACHN cells and verified the knockdown efficiency (Figure S12A). A subcutaneous tumor-bearing model was used to study its effect on tumor proliferation, and a tail vein lung metastasis model and popliteal lymph node metastasis model were used to study its effect on tumor hematogenous and lymphatic metastasis (Figure 8A). In subcutaneous tumor-bearing experiments, treatment with the SC79 reversed the *PRR11* knockdown-induced reductions in tumor volume and weight (Figure 8B-C and Figure S12B), further supporting the establishment of the PRR11-AKT axis. ^18^F-FDG PET/CT is a commonly used tumor test in the clinic and plays an important role in the diagnosis and treatment of a variety of malignant tumors 33. PET/CT imaging showed that tumor activity was significantly reduced in the *PRR11* knockdown group, and SC79 treatment reversed the effect of PRR11 on tumors (Figure 8D). IHC demonstrated that *PRR11* silencing reduced the abundance of pS473-AKT and Ki67 while increasing γ-H2AX levels, and these effects were counteracted by SC79 treatment (Figure 8E and Figure S12C). In addition, we established a tail vein lung metastasis model and found that *PRR11* silencing significantly suppressed the fluorescence intensity and number of metastatic tumor nodules, and this suppression was significantly counteracted by SC79 treatment (Figure 8F-G and Figure S12D). Consistently, the popliteal lymph node metastasis model generated via MRI scanning and dissection also showed that popliteal lymph nodes were smaller in the *shPRR11* group than in the *shNC* group, and this effect was counteracted by SC79 treatment (Figure 8H-I and Figure S12E). H&E staining clearly revealed lymph node tumor infiltrating lesions (Figure S12F). These findings suggest that PRR11 promotes the proliferation and migration of RCC cells by activating the AKT pathway. In conclusion, PRR11 affects oxidative DNA damage by activating the AKT pathway, thus promoting the proliferation and migration of RCC cells.

In summary, we found that PRR11-mediated activation of the AKT pathway affects oxidative DNA damage and accelerates RCC progression via a molecular mechanism in which FBXW7-GSK3β mediates PRR11 degradation. On the other hand, PRR11 activates the AKT signaling pathway, which in turn inhibits GSK3β activity, thereby preventing PRR11 degradation, forming a positive feedback loop and accelerating RCC progression (Figure 8J).

## Discussion

Because RCC cells may exhibit sensitivity to defects in homologous recombination genes, there has been increasing interest in studies related to RCC-associated DNA damage 34. DNA damage and subsequent genomic instability can lead to gene mutations and chromosomal damage, thereby promoting tumor development 35, 36. On the other hand, massive and extensive DNA damage can cause fatal damage to tumor cells by inducing apoptosis or necrosis, cellular senescence, and cell cycle arrest 37, 38. Importantly, oxidative DNA damage caused by ROS is the main cause of DNA damage 39.

In earlier studies, we found that *PRR11* silencing caused cell apoptosis and cell cycle arrest in RCC 7. Interestingly, oxidative DNA damage is also closely related to apoptosis and cell cycle progression 26. Here, for the first time, we elucidated the important role of *PRR11* silencing in causing oxidative DNA damage and thus inhibiting RCC development. Notably, PRR11 is a rapidly degradable protein with multiple ubiquitination and phosphorylation sites and is potentially degraded via the ubiquitin proteasome 9. Based on these findings, we provide compelling evidence that the E3 ligase FBXW7 promotes the phosphorylation-dependent ubiquitination and degradation of PRR11 via a mechanism mediated by GSK3β.

AKT is a major regulator of cell survival that modulates biological functions by phosphorylating a wide range of kinases, enzymes, and transcription factors and is therefore frequently activated as a cancer driver 45, 46. Notably, many RCC tumor inhibitors, including various cellular proteins, inhibit cell growth/migration inhibition and apoptosis by inhibiting the AKT signaling pathway 47-49. GSK3β is regulated by AKT as its substrate, and AKT can inhibit GSK3β activity by phosphorylating the Ser9 site of GSK3β and thus inhibiting GSK3β activity 50. Based on RNA-seq analysis and western blot experiments, we found that *PRR11* amplification overactivated the AKT pathway through down-regulation of PTEN, which in turn affected the expression of signals downstream of the pathway, including the inactivation of GSK3β. Interestingly, GSK3β is involved in FBXW7-mediated PRR11 degradation. Importantly, we further demonstrated that inactivation or activation of the AKT pathway via GSK3β markedly affected the strength of the protein interactions between PRR11 and GSK3β and FBXW7. Based on these findings, we revealed the formation of a positive feedback loop between AKT-GSK3β-PRR11.This mutual activation between PRR11 and AKT is essential for maintaining mutual PRR11 overexpression, which enhances the procarcinogenic effect of PRR11-AKT on RCC.

The inactivation of AKT could impair DNA repair mechanisms, including non-homologous end joining (NHEJ) and homologous recombination (HR), leading to delayed DNA damage repair and ultimately resulting in genomic instability and the accumulation of mutations 51, 52. Notably, *AKT* knockout mice show impaired DNA damage-dependent induction of p21 and increased tissue apoptosis, similar to the DNA-PK deficiency phenotype 53. In addition, AKT inactivation could inhibit the expression of multiple antioxidant enzymes and regulate glutathione metabolism by down-regulating the KEAP1-NRF2 pathway, thereby promoting intracellular ROS accumulation 54-56. Importantly, ROS accumulation could induce DNA damage and affect DNA damage response (DDR), which includes DNA strand breaks, base oxidation, and other forms of DNA structural damage 28. Numerous studies have shown that AKT is an important regulator of genome stability and can modulate oxidative DNA damage 57. For example, AKT regulates the response of p53 to oxidative stress to promote cell proliferation and tumorigenesis 58. In addition, AKT can mediate the activation of protective responses to oxidative DNA damage 59. We modeled AKT activation and inactivation by adding the AKT agonist SC79 and the AKT inhibitor MK2206 and found that AKT activation significantly attenuated the increase in oxidative DNA damage mediated by *PRR11* silencing. Conversely, inactivation of AKT counteracted the PRR11 overexpression-mediated decrease in oxidative DNA damage. Consistent with these findings, we demonstrated that the activation of AKT significantly counteracted the reduction in the proliferation and migration of RCC cells caused by *PRR11* silencing. Our experiments suggested that the PRR11-AKT feedback loop influenced the proliferation and migration of RCC cells by regulating oxidative DNA damage.

However, this study also has some limitations. We demonstrated that the PRR11-AKT positive feedback loop can regulate ROS accumulation and thus affect oxidative DNA damage. However, the exact mechanism of how the PRR11-AKT loop regulates ROS remains to be explored. Furthermore, since the AKT pathway genetically targets more pathway components than any other growth factor signaling pathway 60, it is reasonable to suspect that oxidative DNA damage is only part of its effect on RCC development. Last, we identified and demonstrated by RNA-seq that PRR11 activates the AKT signaling pathway by down-regulating PTEN in RCC, but the specific molecular biological mechanisms still need to be further elucidated in future.

In conclusion, our study revealed that FBXW7-GSK3β mediates PRR11 degradation, thereby regulating the AKT pathway. In turn, AKT-GSK3β is involved in regulating PRR11 degradation, forming a positive feedback loop that regulates oxidative DNA damage and accelerates RCC progression.

## Methods

### Cell culture and transfection

The RCC cell lines (ACHN and Caki-1) and HEK293T cells used in this study were obtained and authenticated by the Chinese Academy of Sciences Cell Bank (Shanghai, China). HEK293T cells were cultured in DMEM, ACHN cells were cultured in MEM and Caki-1 cells were cultured in McCoy's 5A. All cell lines were maintained in medium supplemented with 10% FBS and confirmed to be free of mycoplasma contamination. Lipofectamine 3000 (L3000015, Invitrogen) was used for transfection in this study.

### Antibodies and reagents

Commercial sources provided the reagents, which were employed in accordance with instructions. Table S8 contains an inventory of the antibodies used in this study.

### siRNAs and plasmids

*siPRR11* (#1: 5'-ACGCAGGCCUUAAGGAGAATT-3', #2: 5'-GGCCUUAAGGAGAAAGUUUTT-3'), *siFBXW7* (#1: 5'-GCAUAUGAUUUUAUGGUAATT-3', #2: 5'-UGAUACAUCAAUCCGUGUUUG-3') and *siGSK3β* (5'-ACACGAAAGUGAUUGGAAATT-3') were purchased from GenePharma (Suzhou, China). PRR11 cDNA was inserted into pcDNA3.1-2×Flag and pcDNA3.1-HA vectors. Flag-FBXW7 was generously gifted by Prof. Qing Guoliang from Wuhan University. Flag-GSK3β was purchased from Miaolingbio (Wuhan, China). Myc-FBXW7, Flag-FBXW7 (R479Q), Flag-FBXW7 (R505C) and Flag-FBXW7 (ΔF-box) were constructed based on Flag-FBXW7-WT by standard subcloning. Flag-GSK3β (S9D) and Flag-GSK3β (Y216A) were constructed based on Flag-GSK3β-WT by standard subcloning. HA-PRR11-1A (T287A/S291A), HA-PRR11-2A (T326A/T330A), HA-PRR11-1A/2A (T287A/S291A/T326A/T330A), HA-PRR11-1D (T287D/S291D), HA-PRR11-2D (T326D/T330D), HA-PRR11-1D/2D (T287D/S291D/T326D/T330D), HA-PRR11-1Δ (I286_S291del), HA-PRR11-2Δ (L325_T330del), HA-PRR11-1Δ/2Δ (I286_S291del/L325_T330del), HA-PRR11 (amino acids 1-100), HA-PRR11 (amino acids 101-250) and HA-PRR11 (amino acids 251-360) were constructed based on HA-PRR11-WT.

### Quantitative reverse transcription PCR (qRT-PCR)

The ReverTra Ace qPCR RT Kit (FSQ-101, TOYOBO) was used to synthesize cDNA after RNA was extracted using the HiPure Total RNA Mini Kit (R4111-03, Magen). Next, cDNA was analyzed using iQ SYBR Green Supermix (1725125, Bio-Rad). The sequences of primers used are listed in Table S9.

### Immunofluorescence

After being fixed for 30 min with paraformaldehyde, the RCC cells were cultured for 40 min at 20°C in a buffer consisting of 2% BSA and 0.3% Triton X-100. After the cells were stained with the corresponding primary antibody overnight at 4°C, the cells were treated with DAPI and the fluorescent secondary antibody. Imaging was performed via confocal laser microscopy (Nikon, Japan), and the images were processed using ImageJ software (version 1.52). For the γH2AX foci formation experiments, at least 100 cells per group were evaluated.

### Immunohistochemistry

Tissue microarrays for immunohistochemistry (HKidE180Su02, n = 149) were purchased from Shanghai Outdo Biotech. Formalin fixation, paraffin embedding, section dewaxing, and hydration were applied to the samples in that order. Subsequent procedures included DAB incubation, primary and secondary antibody incubation, and serum blocking.

### Alkaline comet assay

Preheated 0.6% normal melting point agarose was dropped on the frosted surface of the same preheated slide, which was covered with a coverslip and solidified at low temperatures. The cell suspension at a concentration of 3 × 10^6^ cells/ml was mixed with 0.6% low melting point agarose. The mixture was dropped on the first layer of agarose and covered with a coverslip to cool and solidify at low temperature. After removing the coverslips, the cells were lysed in lysis solution (2.5 mM NaCl, 100 mM EDTA, 10 mM Tris, pH = 10) at 4°C for 8 h. Slides containing cells were soaked in electrophoresis buffer (1 mM EDTA, 300 mM NaOH, pH > 13) for 30 min to allow DNA to unwind, followed by electrophoresis in electrophoresis buffer at 300 mA/30 V for 30 min. The slides were washed and neutralized, dehydrated with anhydrous ethanol, and finally stained with DAPI. Images were taken using a fluorescence microscope, and cellular comet tail DNA was quantified using CASP software (version 1.2.3b1). For each case, at least 150 cells were analyzed.

### Flow cytometry

To detect reactive oxygen species (ROS), the collected RCC cells were stained with 10 μM DCFH-DA (D6883, Sigma) in PBS for 20 min, protected from light, and then washed with PBS. Flow cytometry (Beckman Cytoflex, USA) was used to measure ROS levels, and FlowJo software (version 10.8.1) was used to analyze the data.

To detect the MMP, collected RCC cells were stained with JC-1 reagent (C2006, Beyotime), and then samples were measured using flow cytometry.

### Western blot analysis

Cells were lysed at low temperature in a mixture of Roche phosphatase inhibitor, protease inhibitor and RIPA buffer (P0013B, Beyotime) for 40 min, followed by high-speed centrifugation to collect the supernatant. SDS-PAGE gel was used to separate the denatured proteins, which were then electrotransferred to a PVDF membrane. The expression of the corresponding proteins was detected following sequential incubation with primary and secondary antibodies.

### Co-immunoprecipitation (co-IP)

The BeaverBeads^TM^ Protein A/G Immunoprecipitation Kit (22202-100, Beaver) was used for co-IP detection. In brief, cells were treated with MG132 (10 μM, S2619, Selleck) for 6 h prior to collection, followed by preparation of cell lysates with cell lysate buffer containing proteasome inhibitors and Roche phosphatase inhibitors, and then incubated overnight with the target antibody at low temperature. After addition of Protein A/G magnetic beads, the antigen-antibody mixture was mixed at low temperature for 2 h. IP buffer was used to wash the protein-antibody-magnetic bead complex several times. Finally, the immune complex was eluted with 1× SDS buffer and analyzed by Western blotting.

### Cycloheximide (CHX)-chase assay

To monitor the half-life and degradation efficiency of the target proteins, 50 μg/mL CHX (HY-12320, MCE) was added to the cell culture medium and mixed before cells were harvested at various intervals for Western blot analysis. The target protein expression levels were quantitatively analyzed using ImageJ software.

### Ubiquitination assay

For PRR11 ubiquitination assay, cells were transfected with the indicated plasmids. 6 h before the cells were harvested, 10 μM MG132 was added to the cell culture media. Roche phosphatase inhibitor and proteasome inhibitor were added to RIPA buffer before cells were lysed. The next steps were consistent with those of the co-IP analysis.

### *In vitro* phosphorylation assay

Following the methods of Moscat J *et al.* 61, 62, 48 h after the transfection of 293T cells with HA-labeled human PRR11 WT or PRR11 4A (T287A, S291A, T326A, T330A), the cells were lysed with RIPA buffer and HA-PRR11 was immunoprecipitated via magnetic beads containing the HA antibody. The immunoprecipitates were incubated with human active GSK3β (G09-10H, SignalChem) for 1 h at 30°C in kinase assay buffer (25 mM Tris-HCl, 25 mM KCl, 5 mM MgCl_2_, 1 mM DTT) containing 500 μM ATP-γ-S (ab138911, Abcam). The reaction was terminated with 0.1 mM EDTA and incubated with 0.5 mM p-nitrobenzyl mesylate (PNBM, ab138910, Abcam) at 30°C for 60 min. Finally, immunoblotting was performed using anti-thiophosphate antibody (ab92570, Abcam).

### Immunoprecipitation-mass spectrometry (IP-MS) analysis

To identify the binding partners of PRR11, HA-Vector and HA-PRR11 plasmids were transfected into 293T cells for 48 h, and the proteins were then analyzed by co-IP. The samples were separated by SDS-PAGE and stained with a Fast Silver Stain Kit (P0017S, Beyotime). SpecAlly Life Technology Co., Ltd. (Wuhan, China) carried out IP-MS. A tims TOF Pro liquid mass spectrometry system (Bruker, USA) was used for mass spectrometry analysis. MaxQuant (version 2.2.0.0) was used to analyze the raw mass spectral data. The software's integrated Andromeda database search method was used. The database searched was the Human Protein Sequence Database obtained from UniProt.

### RNA-seq and bioinformatics analysis

DNase I extraction of RNA was followed by DNA digestion. A NanoDrop^TM^ OneC Spectrophotometer (Thermo Fisher Scientific, USA) was used to for RNA quality control. 2 μg of RNA was used for stranded RNA sequencing library preparation. PCR products were enriched, quantified, and sequenced on a DNBSEQ-T7 sequencer (MGI Tech, China). Raw data were filtered, low-quality reads were discarded, and reads contaminated with adaptor sequences were trimmed. Clean data were mapped to the human genome (assembly GRCh38) using STAR software (version 2.5.3a), and reads were counted using featureCounts (Subread-1.5.1; Bioconductor).

Based on the TCGA-KIRC dataset, the top 200 genes associated with PRR11 were screened by Pearson correlation analysis. These 200 genes were subsequently analyzed by Gene Ontology (GO) and Kyoto Encyclopedia of Genes and Genomes (KEGG). For the RNA-seq expression matrix, differential expression genes (DEGs) were identified using the R package “edgeR” after setting the screening threshold at *p* < 0.05. Subsequently, gene set enrichment analysis (GSEA) was performed for all genes in the expression matrix and KEGG analysis was performed for DEGs. The R package "clusterProfiler" was used to carry out GO, GSEA, KEGG. As a reference, the annotated gene set c2.cp.kegg.v2023.1.Hs.symbols.gmt was chosen. *p* < 0.05 was considered to indicate statistical significance.

### Protein molecular docking

FBXW7 (UniProt ID: Q969H0) and PRR11 (UniProt ID: Q96HE9) were used as docking protein models. Protein-protein molecular docking was performed using HDOCK server (*http://hdock.phys.hust.edu.cn/*) 63. Protein pre-processing (deletion of water molecules and redundant ligands, addition of hydrogen atoms) was performed and visualization was performed via PyMol 2.4 software. Docking Score, Confidence Score and Ligand RMSD were used as the evaluation criteria for docking and the model with the highest score was selected as the best docking model.

### Proliferation assays

For MTT assay, RCC cells were seeded in 96-well plates at 3000 cells/well. 4 h before the assay, 20 μL MTT (5 mg/mL, HY-15924, MCE) solution was added to each well, the medium was discarded, the cells were dissolved in 150 μL of DMSO and shaken well, and the microplate reader (SpectraMax M2, USA) was used to measure the absorbance.

For colony formation assay, RCC cells were planted in 6-well plates with 1000 cells/well, fixed with formaldehyde after 2 weeks, and stained with crystal violet.

### Migration assays

For transwell assay, 4 × 10^4^ RCC cells were placed in upper chamber (Corning, USA) with serum-free medium, and migration was induced in the bottom chamber with serum-containing medium. The cells were finally fixed and stained.

### AKT kinase activity assay

Briefly, proteins were first quantified using the BCA protein assay kit (P0011, Beyotime). AKT kinase activity was then assayed using the AKT kinase activity assay kit (ADI-EKS-400A, Enzo). AKT kinase assay was performed on 10 μg of proteins from each set of samples according to the manufacturer's instructions, and the absorbance of the reactants at 450 nm was measured by a microplate reader.

### GST pull-down assay

Recombinant GST and His-FBXW7 proteins were purchased from CUSABIO Co., Ltd (Wuhan, China). Human PRR11 was subcloned into the pGEX-6p-1 construct. Recombinant GST tagged PRR11 was purified from *Escherichia coli* BL21. GST-PRR11 fusion protein was incubated with His-FBXW7 and His-GSK3β fusion proteins for 3 h at 4°C, respectively. The mixtures were incubated with glutathione-Sepharose beads for 2 h at 4°C for GST pull-down assay. GST pull-down products were eluted with 1× SDS buffer and denatured at 100°C for 8 min, then used for Western blotting.

### Animal studies

The Laboratory Animal Welfare and Ethics Committee of Zhongnan Hospital of Wuhan University provided consent for all animal experimental procedures (approval number: ZN2022263). Four-week-old male BALB/c nude mice purchased from GemPharmatech Co., Ltd. (Nanjing, China) were acclimatized and fed for one week. Lentivirus from GenePharma (Suzhou, China) was used to transfect ACHN cells, and puromycin (540222, Sigma) was used to screen the stable *PRR11* silencing cells (*shPRR11*) or negative control cells (*shNC*). The mice were randomly divided into four groups: *shNC*+DMSO, *shPRR11*+DMSO, *shNC*+SC79, and *shPRR11*+SC79.

For subcutaneous tumor-bearing experiments, 5 × 10^6^ *shNC* or* shPRR11* ACHN cells/100 μL of PBS were subcutaneously injected into nude mice. After 10 days of tumor growth, DMSO or SC79 (10 mg/kg, HY-18749, MCE) was intraperitoneally injected every 3 days for 8 times. Every 4 days following injection, the tumor size was recorded, and the tumor volume was calculated using the formula V = 1/2 × L × S^2^, where L represents the long diameter and S represents the short diameter. For positron emission tomography computed tomography (PET/CT) imaging, mice were injected with ^18^F-FDG (150 μCi/per mouse) in the tail vein, anesthetized with 2% isoflurane 1 h later, and then subjected to a 10-minute static PET scan using an Inliview-3000B system (Novelmedical, China). Data were reconstructed using a 3D ordered subsets expectation maximization algorithm.

For the lung metastasis model, ACHN cells were injected via the mice tail vein with 1 × 10^6^ cells/100 μL of PBS. DMSO or SC79 was injected intraperitoneally as previously described. The fluorescence intensity of lung metastases in nude mice was measured by IVIS Spectrum Optical *In vivo* Imaging System (PerkinElmer, USA) after 8 weeks of feeding. The radiation efficiency was calculated as (p/sec/cm^2^/sr)/(μW/cm^2^).

For the popliteal lymph node metastasis model, ACHN cells (1 × 10^6^ cells/50 μL of PBS) were injected into the right foot pads of the mice. DMSO or SC79 was added as previously described. Mouse popliteal lymph nodes were imaged using a 7.0T BioSpec 70/20 (Bruker, USA) small animal magnetic resonance imager (MRI). Subsequently, mice were sacrificed, and popliteal lymph nodes were dissected, measured, and fixed.

### Statistical analysis

At least three independent replications of each experiment were conducted. The data used for the statistical analysis were analyzed with GraphPad Prism 8. The two-tailed Student's t-test was used to compare the two groups, and one-way ANOVA was used to evaluate multiple group differences, Tukey test was used to compare all column pairs, and Dunnett test was used to compare all columns with control column. *p* < 0.05 was considered to indicate statistical significance.

## Supplementary Material

Supplementary figures and tables, dataset descriptions.

Supplementary datasets.
