# Supplementary material for: FBXW7/GSK3β-mediated proline-rich 11 degradation promotes oxidative DNA damage and inhibits tumor progression in renal cell carcinoma
Source: Theranostics. 2025 Feb 3;15(7):2814–33. doi: 10.7150/thno.106018 (PMC11898301; doi:10.7150/thno.106018)
Supplement: Supplementary file 1 — Supplementary figures and tables, dataset descriptions. [file thnov15p2814s1.pdf]

Supplementary Materials

FBXW7/GSK3β-mediated proline-rich 11 degradation promotes oxidative DNA damage and inhibits tumor progression in renal cell carcinoma

Supplementary Figures S1-S12: Pages 2-15

Supplementary Tables S1-S9: Pages 16-27

Description of Supplementary Datasets S1-S5: Page 28

Supplementary Figures

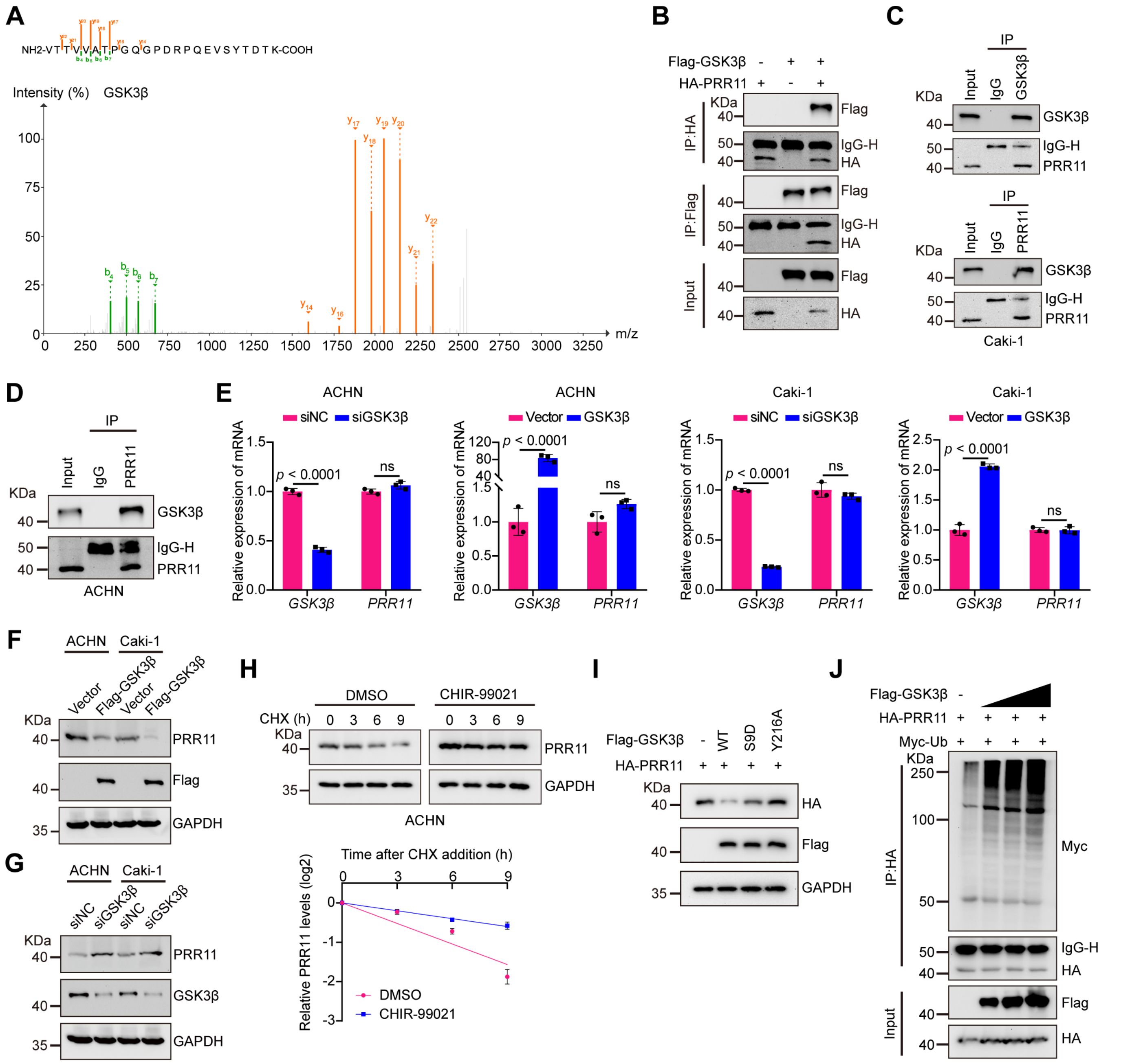

Figure S1. GSK3β interacts with PRR11 and affects PRR11 degradation.
(A) Analysis of the ability of the GSK3β peptide to interact with the PRR11 protein by mass spectrometry. **(B)** 293T cells were transfected with Flag-GSK3β and HA-PRR11 plasmids, Western blot analysis of HA-PRR11 and Flag-GSK3β was performed after IP analysis with HA and Flag antibodies. **(C)** Western blot analysis of PRR11 and GSK3β in Caki-1 cells after IP analysis with antibodies against GSK3β (top) and PRR11 (bottom). **(D)** Western blot analysis of GSK3β in ACHN cells after IP analysis with antibodies against PRR11. **(E)** *GSK3β* was silenced or overexpressed in RCC cells, the transcription levels of GSK3β and PRR11 were verified by qRT-PCR analysis (n = 3 biologically independent experiments). **(F-G)** RCC cells were transfected with Flag-GSK3β or *siGSK3β*, and PRR11 protein expression was verified by Western blot analysis. **(H)** After exposing ACHN cells treated with DMSO or CHIR-99021 (10 μM) to CHX (50 μg/mL) for the indicated durations, Western blot analysis was conducted for PRR11 (top). Quantification of the PRR11 half-life (bottom, n = 3 biologically independent experiments). **(I)** Western blot analysis of HA-PRR11 after transfection of

293T cells with HA-PRR11, Vector, Flag-GSK3β-WT, or Flag-GSK3β-Mut (GSK3β-S9D and GSK3β-Y216A). **(J)** 293T cells were transfected with increasing concentrations of Flag-GSK3β (0.2 μg, 1.0 μg, or 4.0 μg), HA-PRR11 and Myc-Ub were incubated with MG132 (10 μM) for 6 h, and Western blot analysis of Myc-Ub was performed after IP analysis with HA antibody. Protein levels were quantitatively detected with ImageJ software, and linear regression was used to analyze the protein half-life (H). The *p*-values were calculated with two-tailed Student's t-test (E). ns: not significant ($p > 0.05$). Data are presented as mean values ± SD.

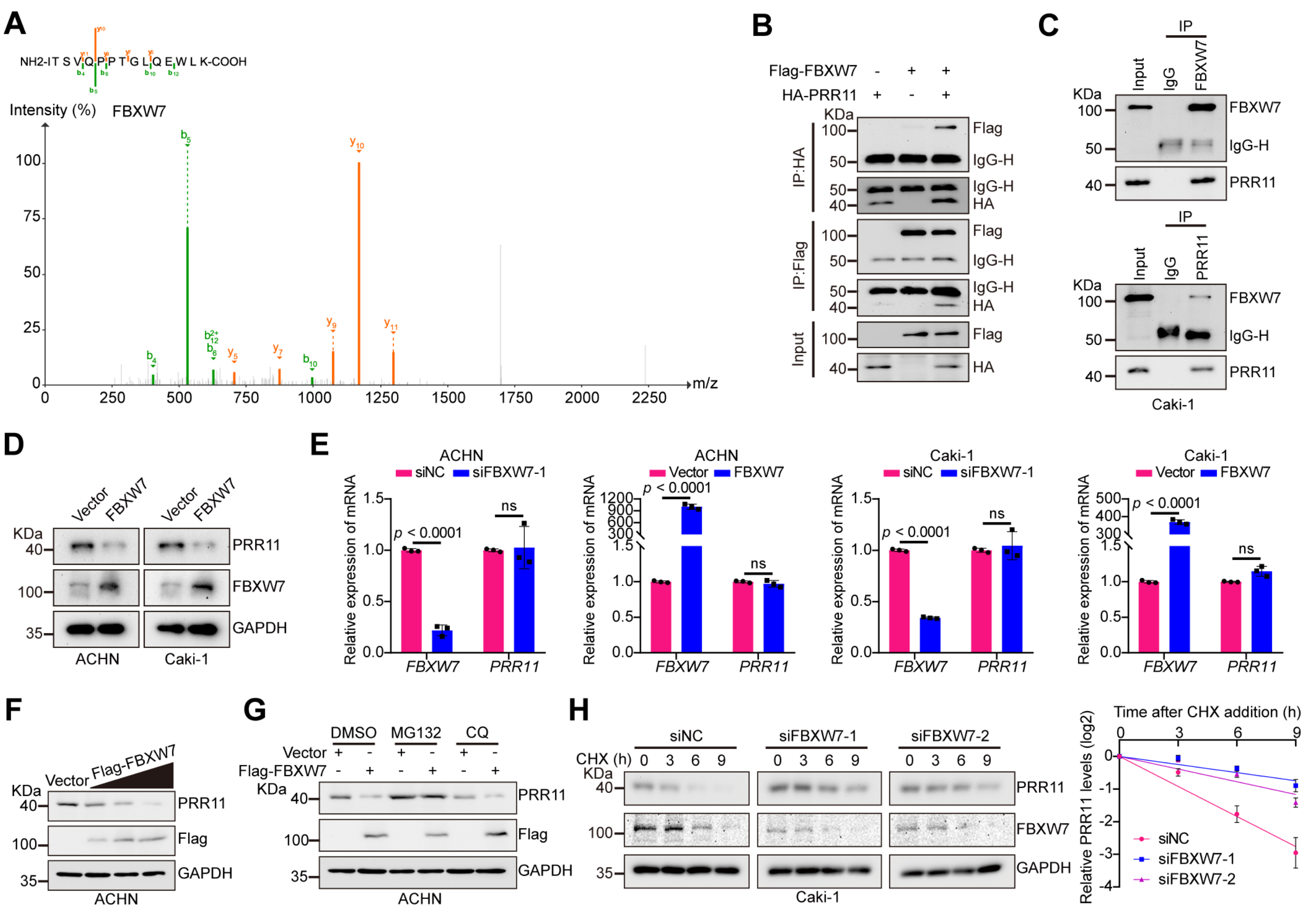

Figure S2. FBXW7 interacts with PRR11 to promote PRR11 degradation.

(A) Analysis of the FBXW7 peptide, a potential PRR11-interacting protein, by mass spectrometry. **(B)** Flag-FBXW7 and HA-PRR11 were transfected into 293T cells, Western blot analysis of HA-PRR11 and Flag-FBXW7 was performed after IP analysis with HA and Flag antibodies. **(C)** Western blot analysis of PRR11 and FBXW7 in Caki-1 cells after IP analysis with FBXW7 (top) and PRR11 (bottom) antibodies. **(D)** Western blot analysis of PRR11 expression in RCC cells after FBXW7 overexpression. **(E)** *FBXW7* was silenced or overexpressed in RCC cells, the transcription levels of FBXW7 and PRR11 were verified by qRT-PCR analysis (n = 3 biologically independent experiments). **(F)** Western blot analysis of PRR11 after the transfection of ACHN cells with Vector or Flag-FBXW7 (0.2 µg, 1.0 µg, or 4.0 µg). **(G)** ACHN cells transfected with Vector or Flag-FBXW7 were incubated with DMSO, MG132 (10 µM), or chloroquine (CQ, 20 µM) for 6 h, after which PRR11 expression was analyzed by Western blot. **(H)** Caki-1 cells transfected with *siFBXW7-1* or *siFBXW7-2* were treated with CHX (50 µg/mL) for the indicated durations, after which Western blot analysis of PRR11 expression was performed (left). PRR11 half-life quantification (right, n = 3 biological independent experiments). Protein levels were quantitatively detected with ImageJ software, and linear regression was used to analyze the protein half-life (H). The *p*-values were calculated with two-tailed Student's t-test (E). ns: not significant ($p > 0.05$). Data are presented as mean values ± SD.

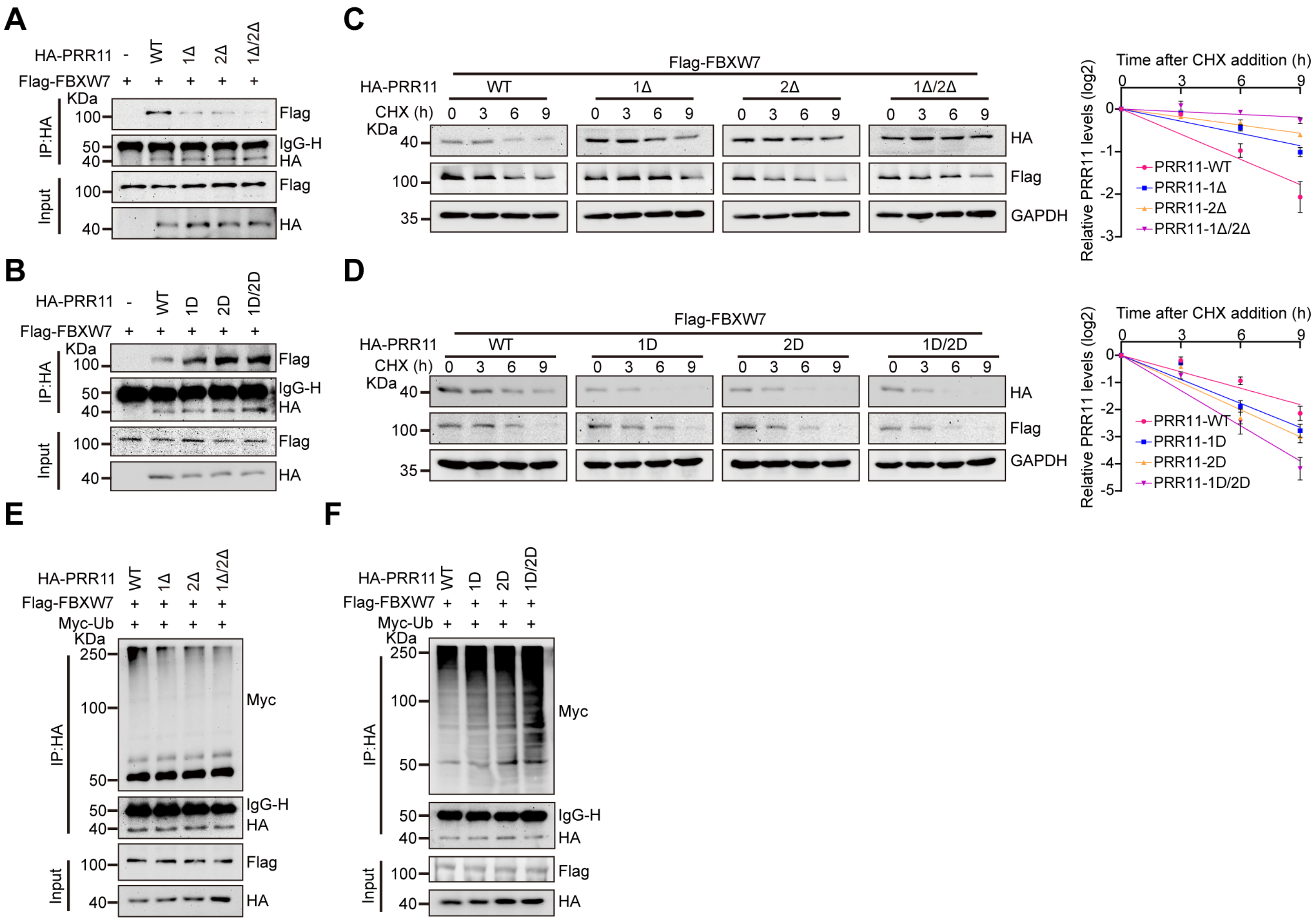

Figure S3. CPD motif deletion in PRR11 disrupts the binding and degradation of PRR11 by FBXW7. **(A)** Flag-FBXW7, Vector, HA-PRR11-WT, or PRR11 CPD motif deletion mutant (PRR11-1Δ, PRR11-2Δ, PRR11-1Δ/2Δ) plasmids were transfected into 293T cells. After IP analysis with an HA antibody, Western blot analysis of Flag-FBXW7 was performed. **(B)** Flag-FBXW7, Vector, HA-PRR11-WT, or PRR11 phosphorylation mimic mutants (HA-PRR11-1D, HA-PRR11-2D, and HA-PRR11-1D/2D) plasmid were transfected into 293T cells. After IP analysis with HA antibody, Western blot analysis of Flag-FBXW7 was performed. **(C)** 293T cells transfected with Flag-FBXW7, HA-PRR11-WT, or PRR11 CPD motif deletion mutants were treated with CHX (50 µg/mL) for the indicated durations, and then Western blot analysis was performed for HA-PRR11 (left). Quantification of the PRR11 half-life (right, n = 3 biologically independent experiments). **(D)** 293T cells transfected with Flag-FBXW7, HA-PRR11-WT, or PRR11 phosphorylation mimic mutants were treated with CHX (50 µg/mL) for indicated times, and then Western blot analysis was performed for HA-PRR11 (left). Quantification of PRR11 half-life (right, n = 3 biologically independent experiments). **(E)** 293T cells transfected with Flag-FBXW7, Myc-Ub, HA-PRR11-WT, or PRR11 CPD motif deletion mutants were incubated with MG132 (10 µM) for 6 h, and Western blot analysis of Myc-Ub was performed after IP analysis with HA antibody. **(F)** 293T cells transfected with Flag-FBXW7, Myc-Ub, HA-PRR11-WT, or PRR11 phosphorylation mimic mutants were incubated with MG132 (10 µM) for 6 h, and Western blot analysis of Myc-Ub was performed after IP analysis with HA antibody. Protein levels were quantitatively detected with ImageJ software, and linear regression was used to analyze the protein half-life (C-D). Data are presented as mean values ± SD.

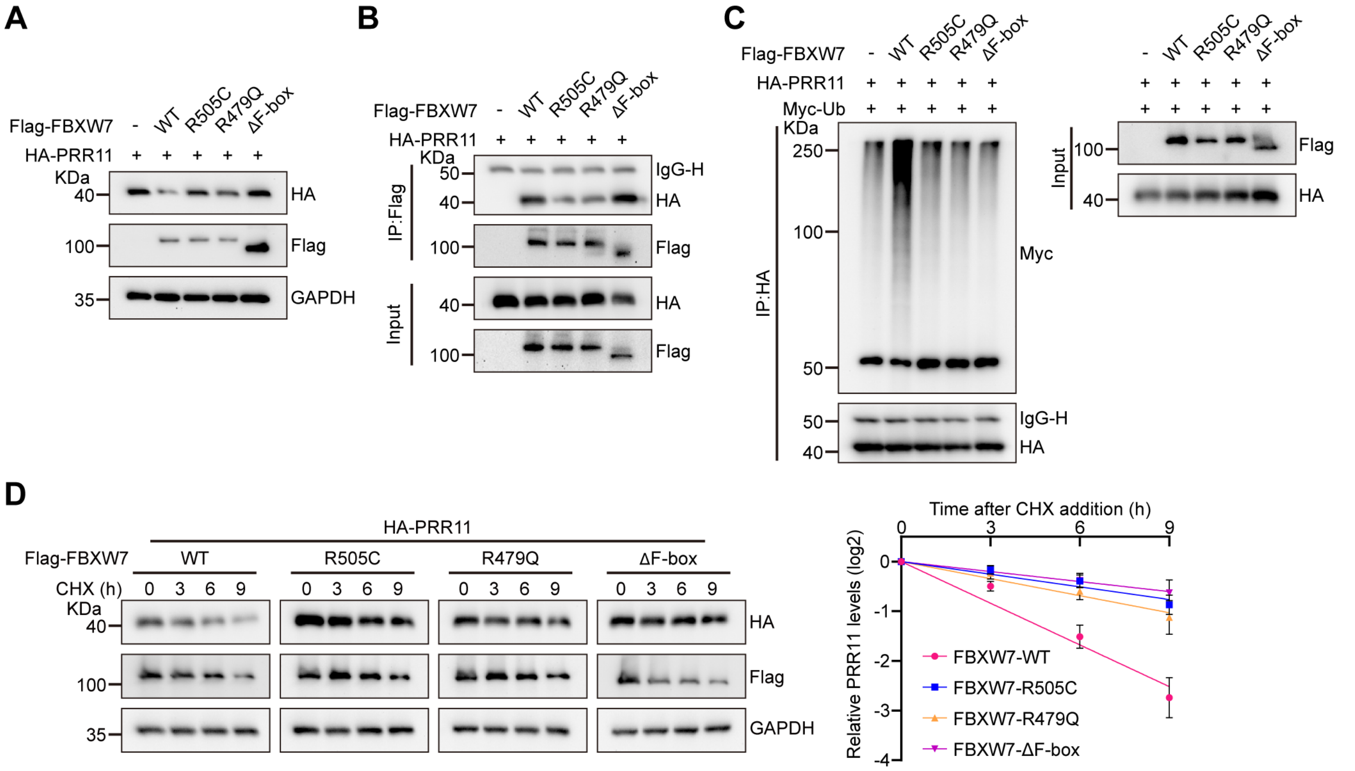

Figure S4. FBXW7 hotspot mutation disrupts FBXW7 binding and the degradation of PRR11.
(A) Western blot analysis of HA-PRR11 after transfection of 293T cells with HA-PRR11, Vector, Flag-FBXW7-WT, or Flag-FBXW7-Mut (FBXW7-R505C, FBXW7-R479Q, and FBXW7-ΔF-box). **(B)** HA-PRR11, Vector, Flag-FBXW7-WT, or Flag-FBXW7-Mut plasmids were transfected into 293T cells. After IP analysis with Flag antibody, Western blot analysis of HA-PRR11 was performed. **(C)** 293T cells transfected with HA-PRR11, Myc-Ub, Vector, Flag-FBXW7-WT, or Flag-FBXW7-Mut plasmids were incubated with MG132 (10 μM) for 6 h, and Western blot analysis of Myc-Ub was performed after IP analysis with HA antibody. **(D)** 293T cells transfected with the HA-PRR11, Flag-FBXW7-WT, or Flag-FBXW7-Mut plasmid were treated with CHX (50 μg/mL) for the indicated durations, after which Western blot analysis was performed for HA-PRR11 (left). Quantification of the PRR11 half-life (right, n = 3 biologically independent experiments). Protein levels were quantitatively detected with ImageJ software, and linear regression was used to analyze the protein half-life (D). The data are presented as the means ± SD.

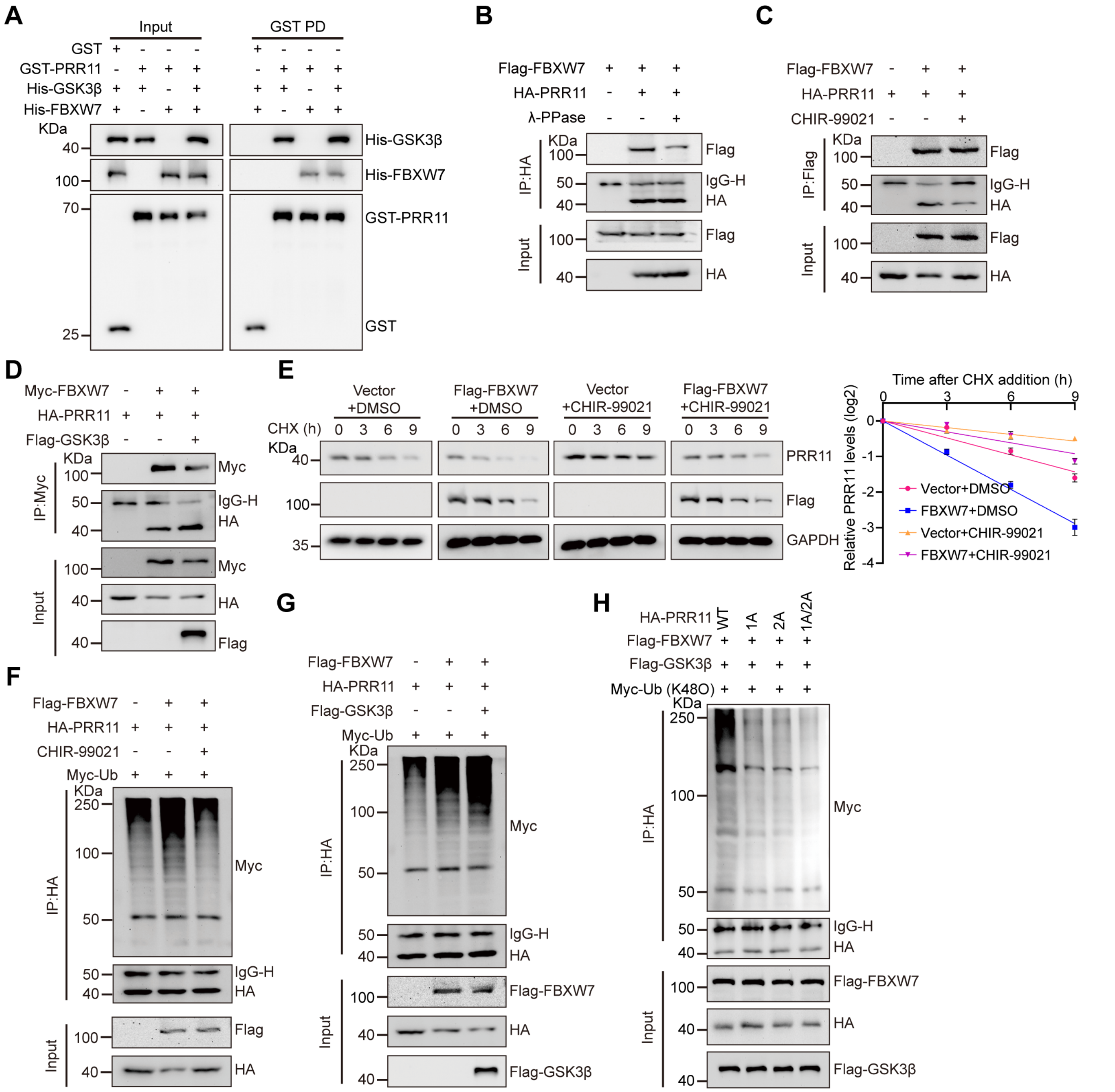

Figure S5. FBXW7 destabilizes PRR11 via GSK3β-mediated phosphorylation.

(A) Western blot analysis of His- GSK3β, His-FBXW7, GST-PRR11 after GST pull-down assays. **(B)** 293T cells transfected with HA-PRR11 or Flag-FBXW7 were treated with λ-PPase. After IP analysis with an HA antibody, Western blot analysis of Flag-FBXW7 was performed. **(C)** 293T cells transfected with Flag-FBXW7 or HA-PRR11 were treated with the GSK3β inhibitor CHIR-99021 (10 μM). After IP analysis with Flag antibody, Western blot analysis was performed on HA-PRR11. **(D)** HA-PRR11, Myc-FBXW7, or Myc-FBXW7+Flag-GSK3β were transfected into 293T cells. After IP analysis with Myc antibody, Western blot analysis was performed on HA-PRR11. **(E)** 293T cells transfected with Flag-FBXW7 were treated with DMSO or CHIR-99021 and incubated with CHX (50 μg/mL) for indicated times, followed by Western blot analysis of PRR11 (left). Quantification of PRR11 half-life (right, n = 3 biologically independent experiments). **(F)** 293T cells transfected with Myc-Ub, Flag-FBXW7, or HA-PRR11 were treated with CHIR-99021 (10 μM). The cells were incubated with MG132 (10 μM) for 6 h before harvest, and Western blot analysis of Myc-

Ub was performed after IP analysis with HA antibody. **(G)** 293T cells transfected with HA-PRR11, Myc-Ub, Flag-FBXW7, or Flag-FBXW7+Flag-GSK3β were treated with MG132 (10 μM) for 6 h, and Western blot analysis of Myc-Ub was performed after IP analysis with HA antibody. **(H)** 293T cells transfected with Myc-Ub (K48O), Flag-FBXW7, Flag-GSK3β, HA-PRR11-WT, or PRR11 dephosphorylation mimic mutants were treated with MG132 (10 μM) for 6 h, and Western blot analysis of Myc-Ub was performed after IP analysis with HA antibody. Protein levels were quantitatively detected with ImageJ software, and linear regression was used to analyze the protein half-life (E).

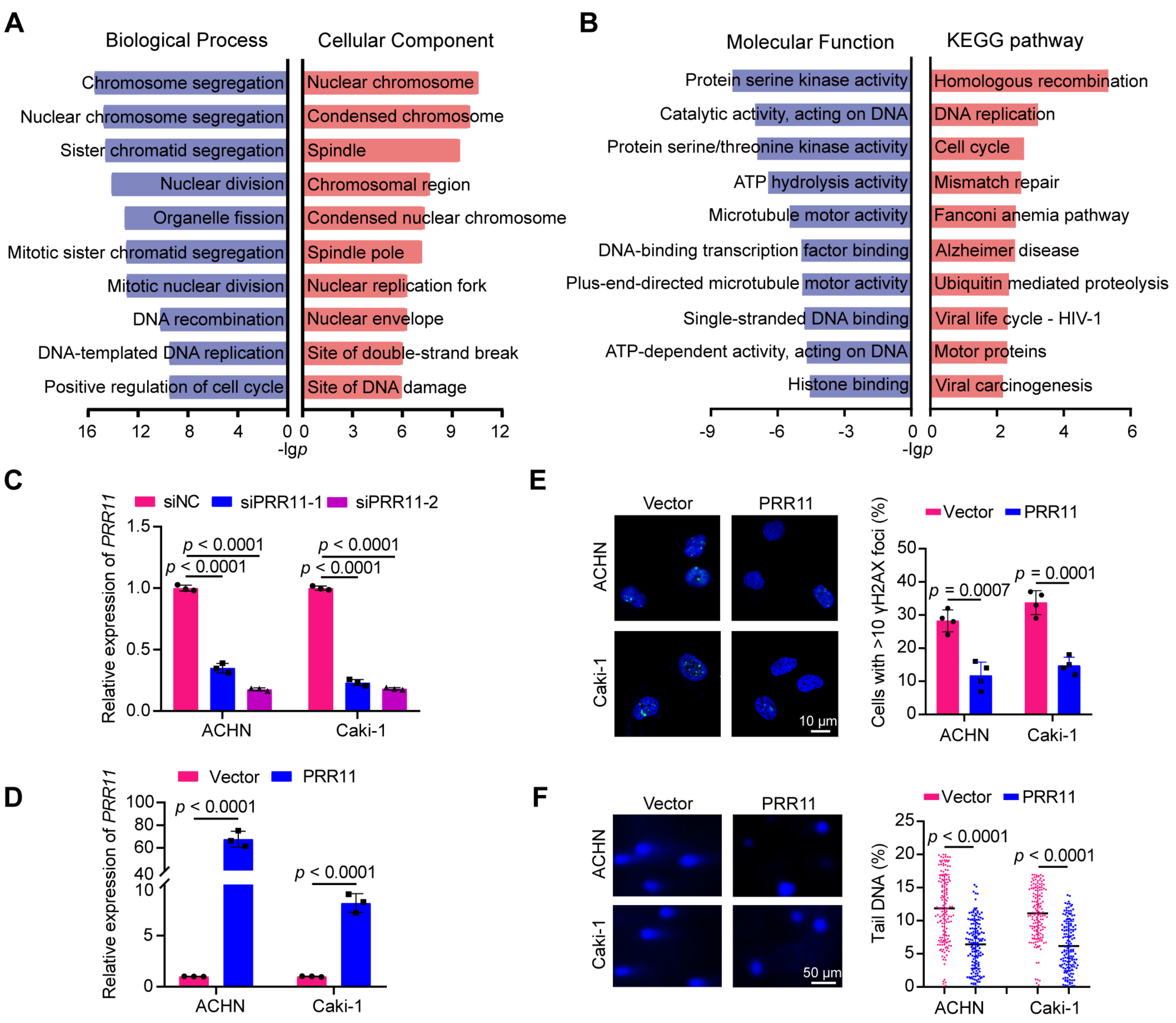

Figure S6. *PRR11* silencing promotes oxidative DNA damage in RCC.

(A-B) GO biological process, cellular component, molecular function and KEGG enrichment analyses of the top 200 genes associated with PRR11 were screened by Pearson correlation analysis based on TCGA database. The *p*-value was calculated using a two-tailed Fisher's exact test. The top 10 most significant pathways were selected based on the p.adjust value and sorted in descending order. **(C-D)** *PRR11* was silenced and overexpressed in RCC cells, and *PRR11* transcript expression was verified by qRT-PCR analysis (n = 3 biologically independent experiments). **(E)** γH2AX staining after PRR11 overexpression in ACHN and Caki-1 cells was analyzed by immunofluorescence (left), and the number of γH2AX foci was quantified (right, n = 4 biologically independent experiments). **(F)** Representative images of alkaline comet assays after PRR11 overexpression in ACHN and Caki-1 cells (left). ACHN and Caki-1 cells per group (n = 150) were quantified using CASP software (right, n = 3 biologically independent experiments). The *p*-values were calculated with one-way ANOVA with Dunnett's multiple comparisons test (C) and two-tailed Student's t-test (D-F). Data are presented as mean values ± SD.

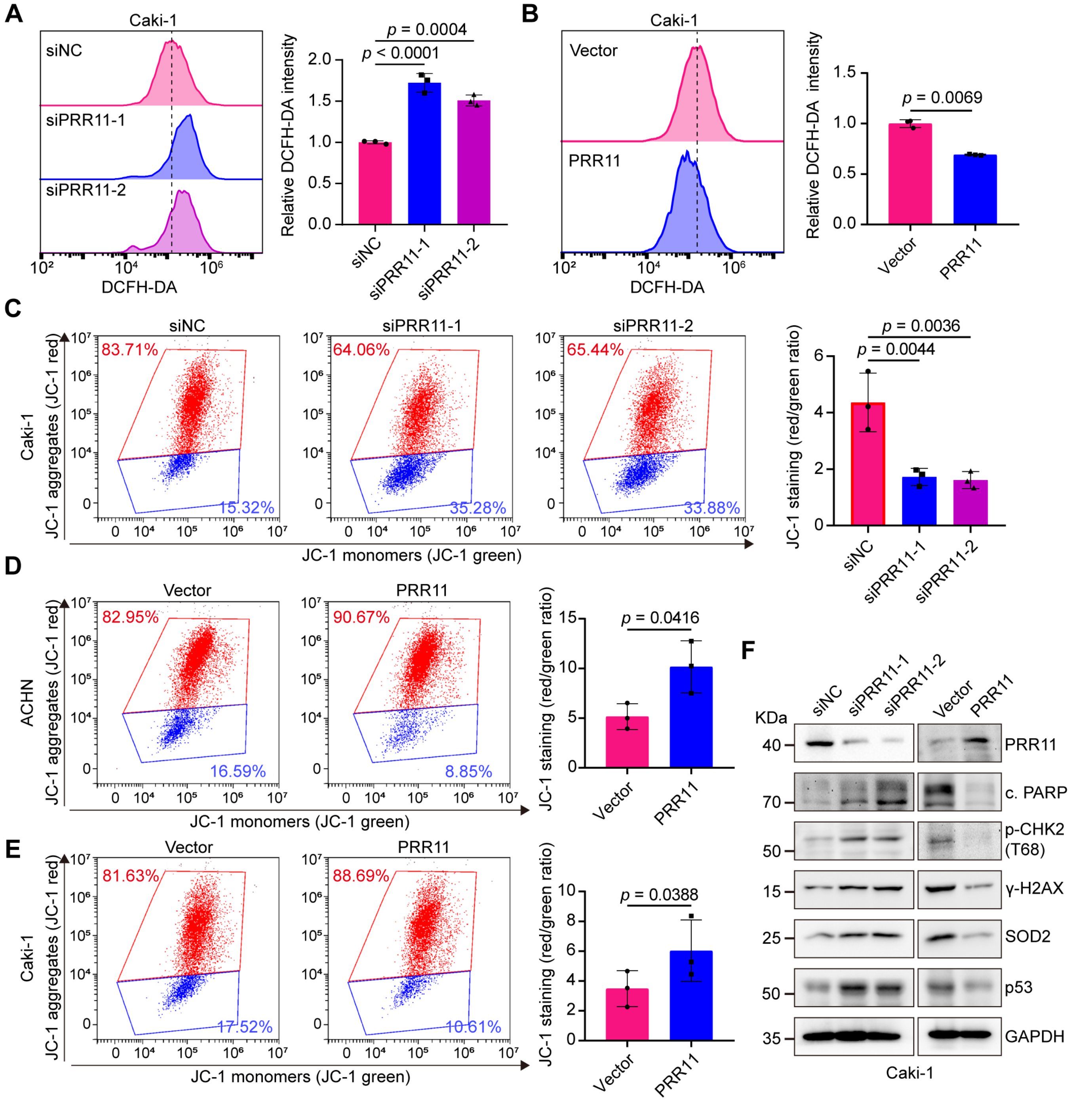

Figure S7. PRR11 is involved in the regulation of ROS and MMP levels.

(A-B) ROS levels in Caki-1 cells following *PRR11* knockdown or overexpression were detected by flow cytometry (left), followed by statistical analysis (right, n = 3 biologically independent experiments). **(C)** Flow cytometry detection of the MMP in Caki-1 cells after *PRR11* silencing (left), followed by statistical analysis (right, n = 3 biologically independent experiments). **(D-E)** Flow cytometry detection of the MMP in RCC cells after PRR11 overexpression (left), followed by statistical analysis (right, n = 3 biologically independent experiments). **(F)** Western blot analysis of DNA damage markers and oxidative stress markers after *PRR11* silencing or overexpression in Caki-1 cells. The *p*-values were calculated with one-way ANOVA with Dunnett's multiple comparisons test (A, C) and two-tailed Student's t-test (B, D-E). Data are presented as mean values ± SD.

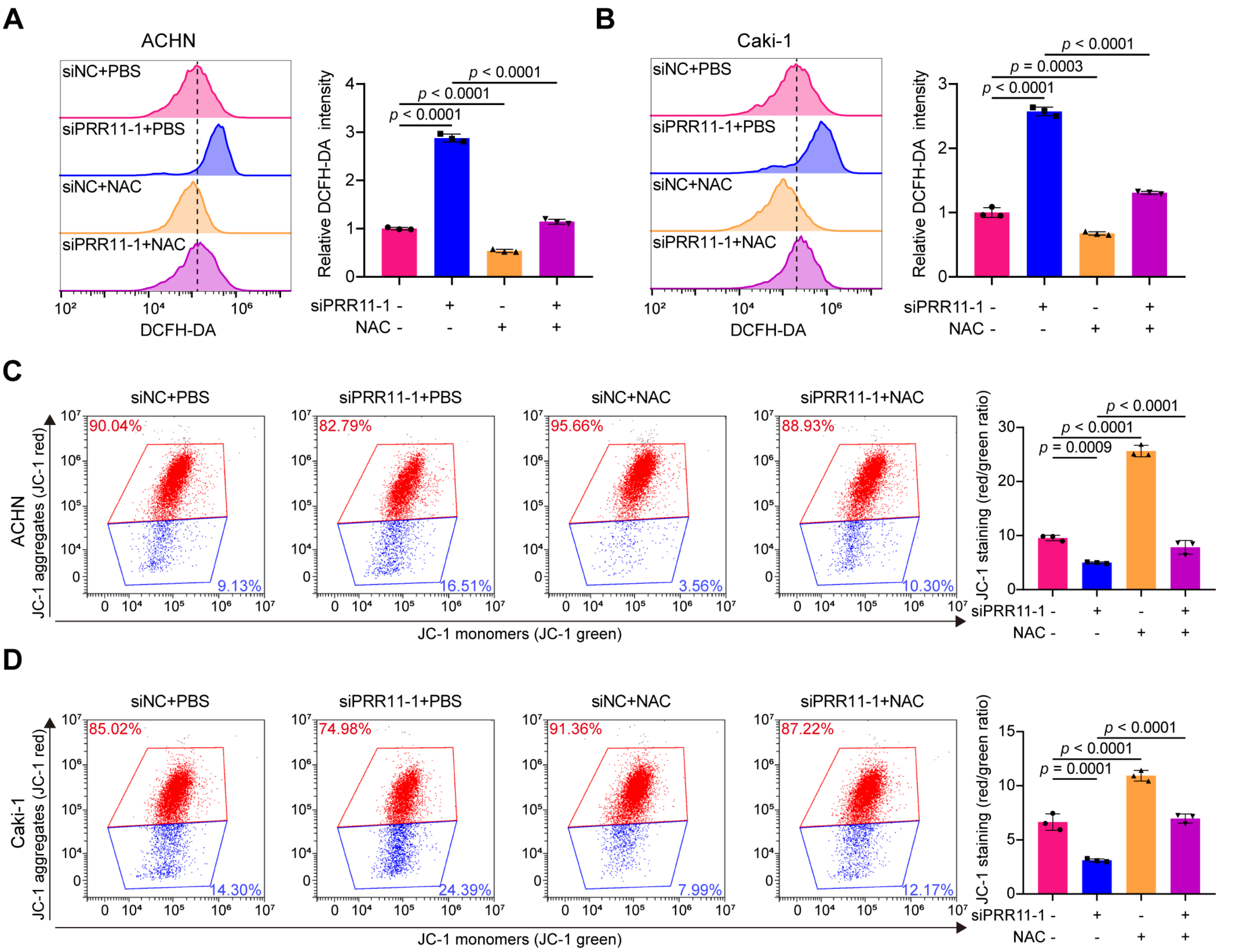

Figure S8. *PRR11* knockdown affects MMP through ROS accumulation.

(A-B) Flow cytometry detection of ROS levels from the indicated groups with *PRR11* silencing or/and ROS scavenger NAC (5 mM) in ACHN and Caki-1 cells (left), followed by statistical analysis (right, n = 3 biologically independent experiments). **(C-D)** Flow cytometry detection of MMP levels from the indicated groups with *PRR11* silencing or/and ROS scavenger NAC (5 mM) in ACHN and Caki-1 cells (left), followed by statistical analysis (right, n = 3 biologically independent experiments). The *p*-values were calculated with one-way ANOVA with Tukey's multiple comparisons test (A-D). Data are presented as mean values ± SD.

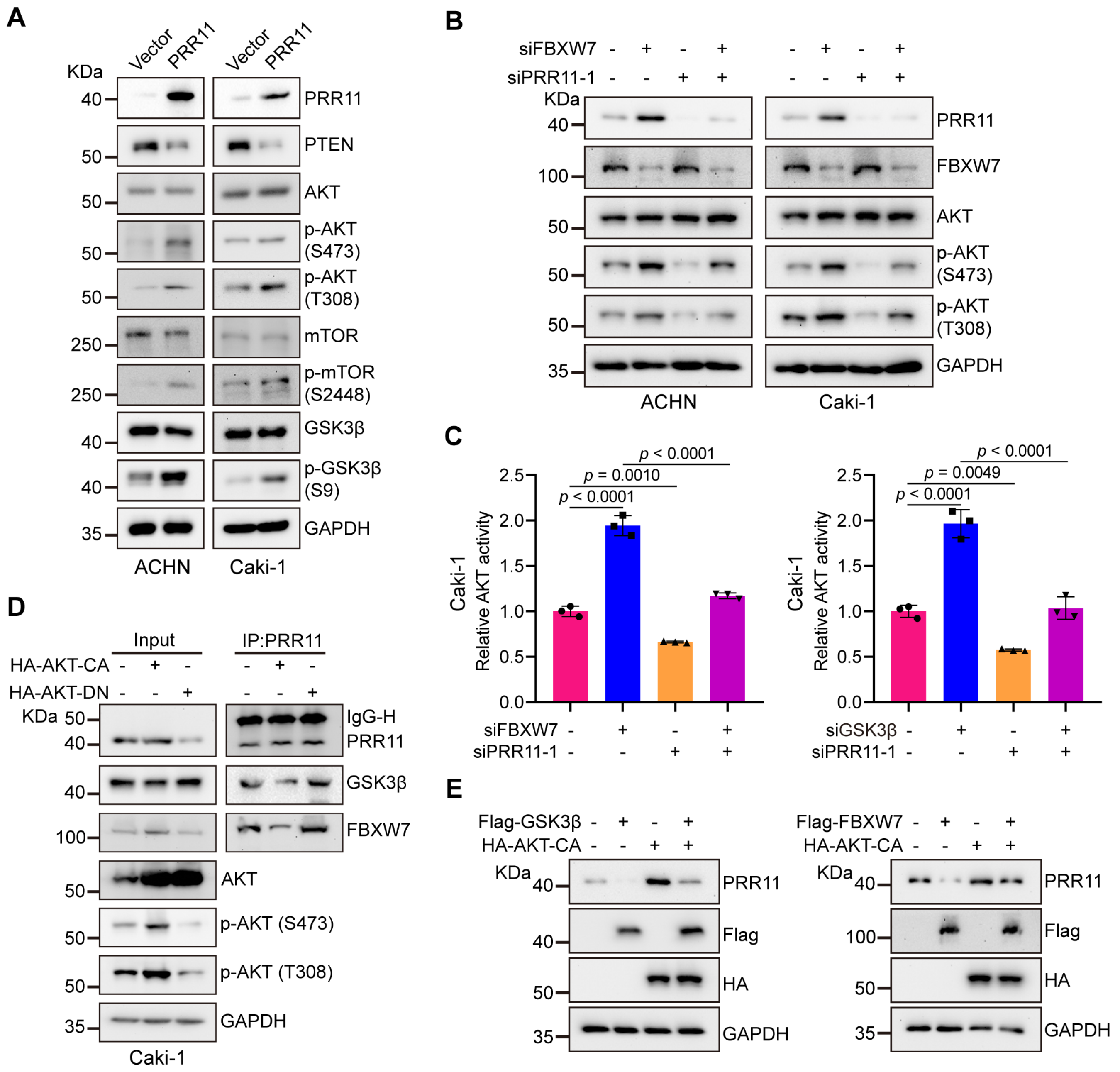

Figure S9. FBXW7/GSK3β-PRR11 axis activates the AKT pathway and AKT activation inhibits PRR11 degradation.

(A) Protein levels of each important factor of the AKT signaling pathway were analyzed by Western blot analysis after PRR11 overexpression in RCC cells. **(B)** Western blot analysis of AKT phosphorylation levels after transfection of ACHN and Caki-1 cells with *siFBXW7* or/and *siPRR11*. **(C)** AKT activity was measured in Caki-1 cells transfected with *siFBXW7* (left)/*siGSK3β* (right) or/and *siPRR11* (n = 3 biologically independent experiments). **(D)** AKT-CA or AKT-DN plasmid was transfected into Caki-1 cells. After IP analysis with PRR11 antibody, Western blot analysis was performed on GSK3β and FBXW7. **(E)** Western blot analysis of PRR11 after transfection of 293T cells with Flag-GSK3β ((left)/Flag-FBXW7 (right) or/and HA-AKT-CA. The *p*-values were calculated with one-way ANOVA with Tukey's multiple comparisons test (C). Data are presented as mean values ± SD.

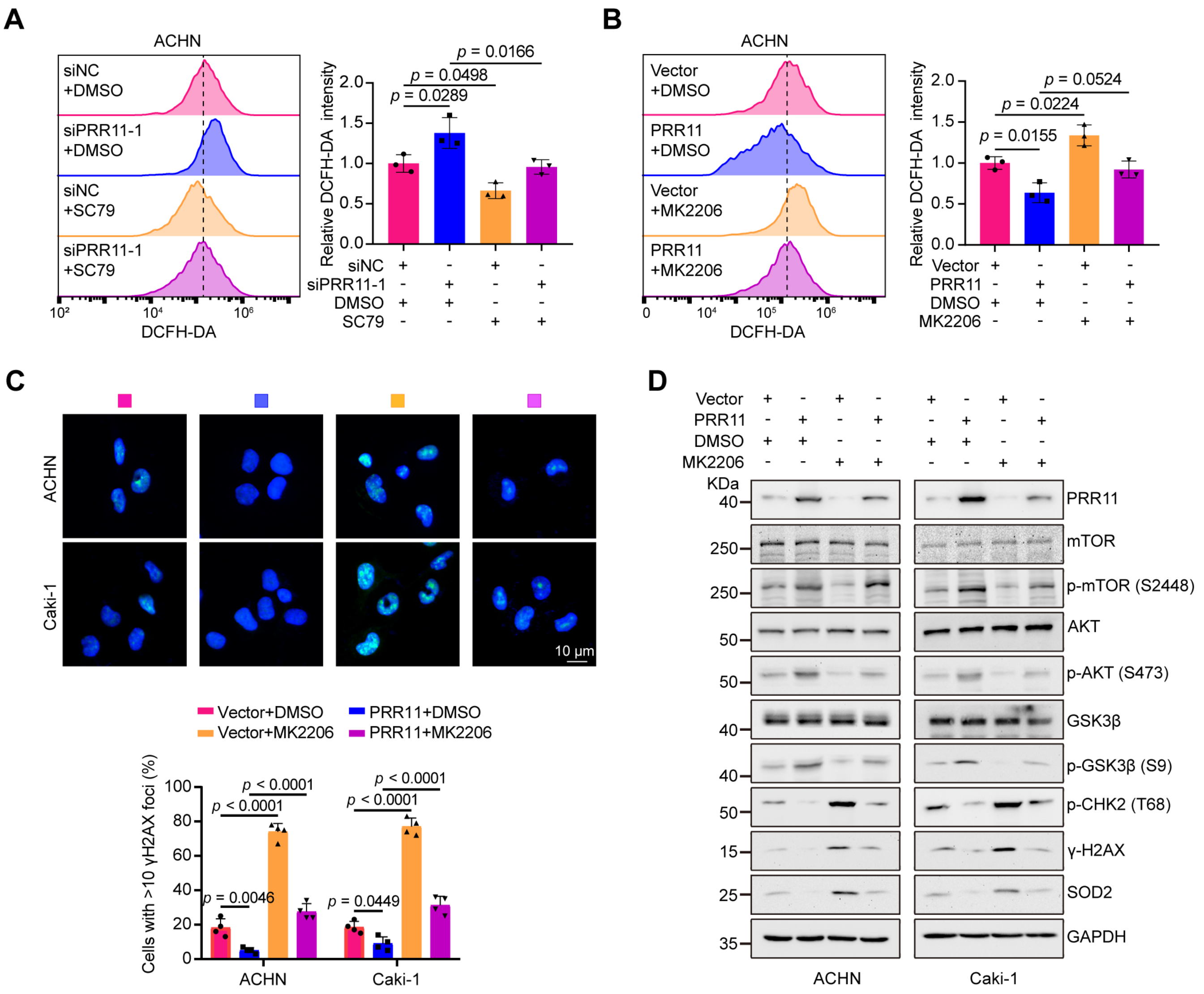

Figure S10. PRR11-AKT axis regulates oxidative DNA damage.

(A) Flow cytometry detection of ROS levels in ACHN cells from the indicated groups with or without *PRR11* silencing and/or treatment with the AKT agonist SC79 (left), followed by statistical analysis (right, n = 3 biologically independent experiments). **(B)** Flow cytometry detection of ROS levels in the indicated groups of ACHN cells treated with or without PRR11 overexpression and/or the AKT inhibitor MK2206 (left), followed by statistical analysis (right, n = 3 biologically independent experiments). **(C)** Immunofluorescence analysis of the number of γH2AX foci in RCC cells with or without PRR11 overexpression and/or MK2206 treatment (top), followed by statistical analysis (bottom, n = 4 biologically independent experiments). **(D)** Western blot analysis of oxidative DNA damage and AKT pathway-associated protein levels in RCC cells with or without PRR11 overexpression and/or MK2206 treatment. The *p*-values were calculated with one-way ANOVA with Tukey's multiple comparisons test (A-C). Data are presented as mean values ± SD.

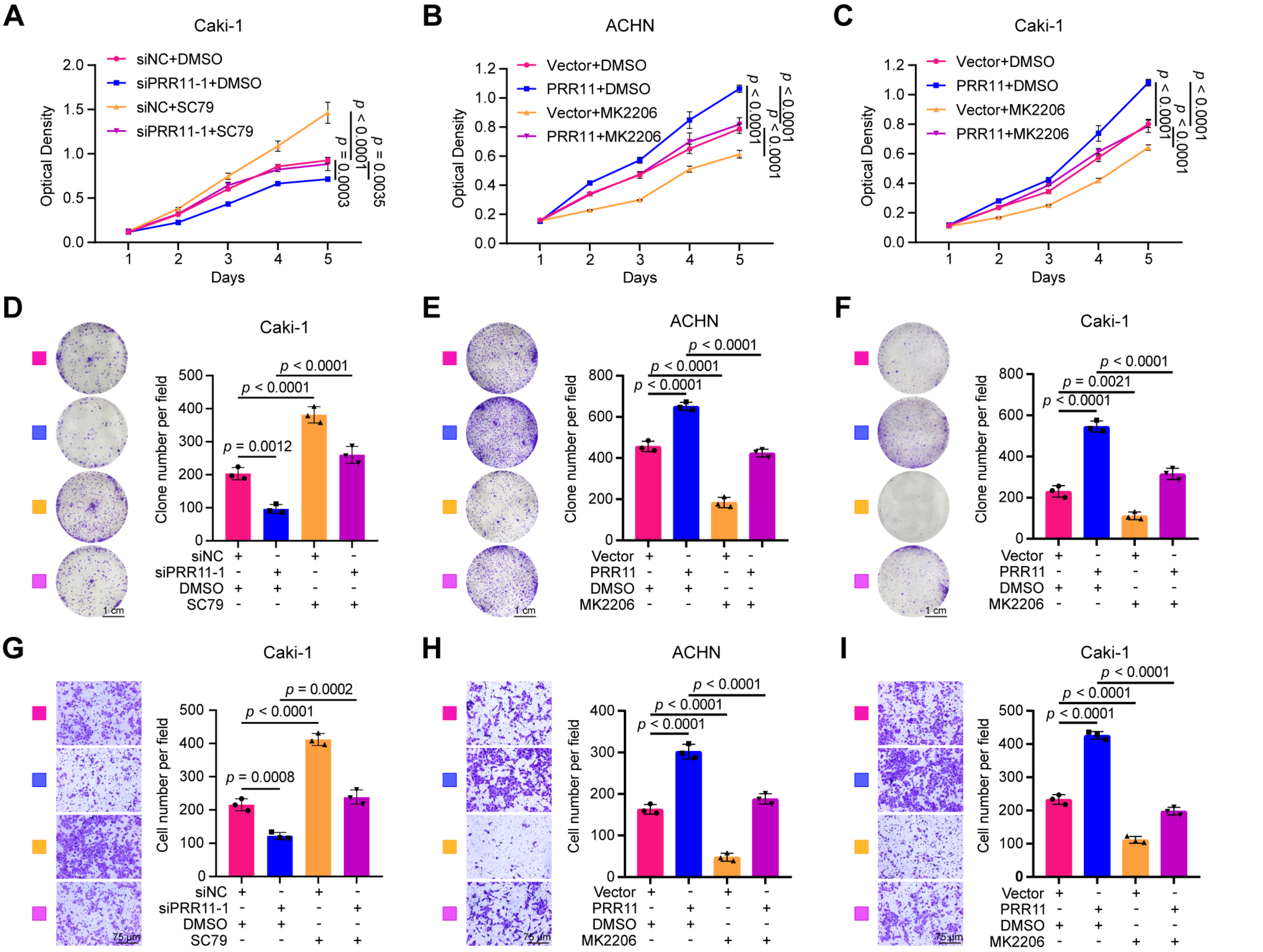

Figure S11. PRR11-AKT axis regulates RCC proliferation and migration *in vitro*.
(A) MTT assay of the proliferative capacity of Caki-1 cells treated with or without *PRR11* silencing and/or AKT agonist SC79 treatment (n = 6 biologically independent experiments). **(B-C)** MTT assay of the proliferative capacity of ACHN and Caki-1 cells treated with or without PRR11 overexpression and/or AKT inhibitor MK2206 treatment (n = 6 biologically independent experiments). **(D)** Colony formation assay of the proliferative capacity of Caki-1 cells treated with or without *PRR11* silencing and/or AKT agonist SC79 treatment (left), followed by statistical analysis (right, n = 3 biologically independent experiments). **(E-F)** Colony formation assay of the proliferative capacity of ACHN and Caki-1 cells treated with or without PRR11 overexpression and/or AKT inhibitor MK2206 treatment (left), followed by statistical analysis (right, n = 3 biologically independent experiments). **(G)** Transwell assay showing the migration capacity of Caki-1 cells treated with or without *PRR11* silencing and/or AKT agonist SC79 treatment (left), followed by statistical analysis (right, n = 3 biologically independent experiments). **(H-I)** Transwell assay showing the migration capacity of ACHN and Caki-1 cells treated with or without PRR11 overexpression and/or AKT inhibitor MK2206 treatment (left), followed by statistical analysis (right, n = 3 biologically independent experiments). The *p*-values were calculated with one-way ANOVA with Tukey's multiple comparisons test (A-I). Data are presented as mean values ± SD.

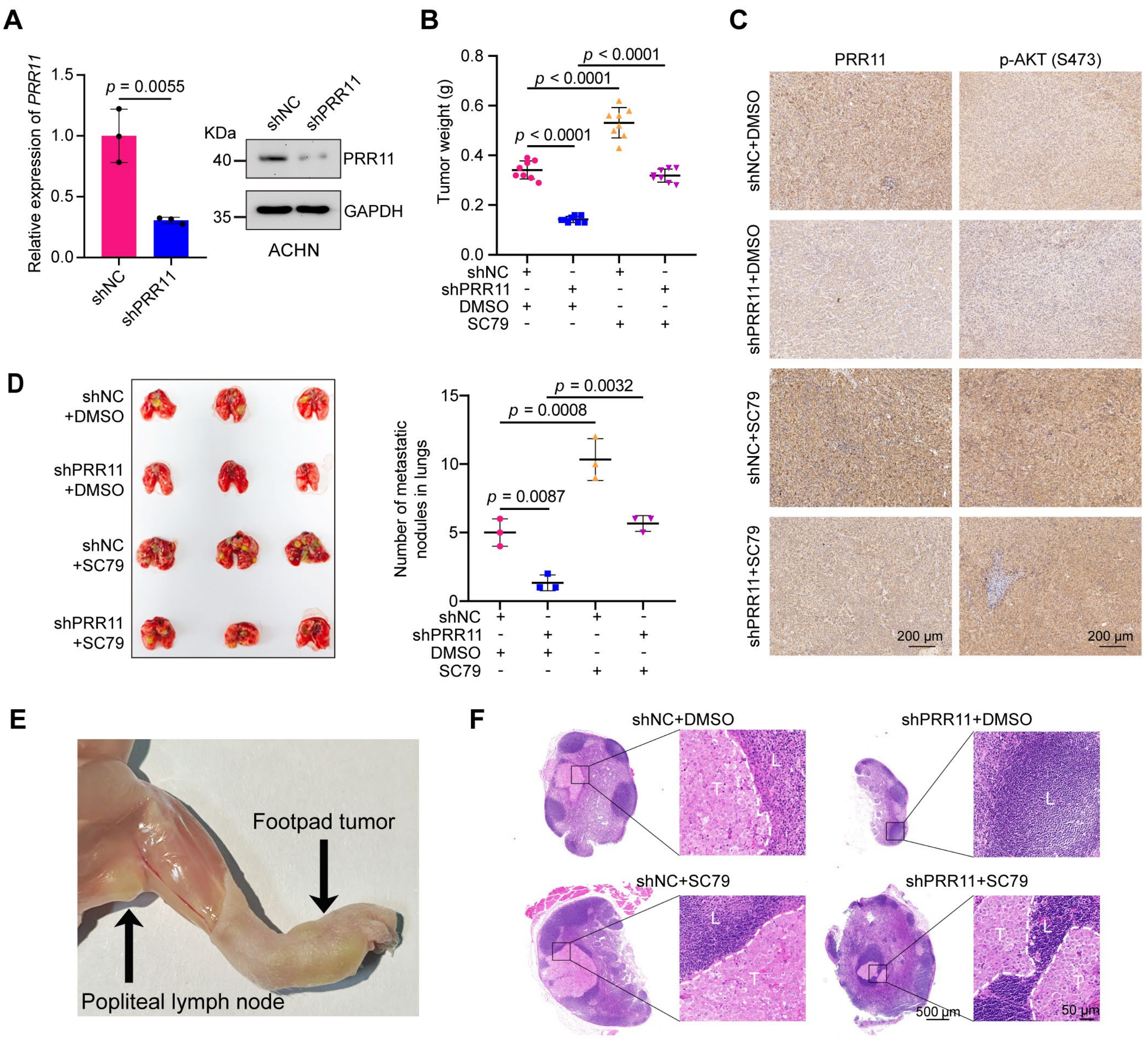

Figure S12. The PRR11-AKT axis promotes RCC proliferation and metastasis *in vivo*.
(A) qRT-PCR (left, n = 3 biologically independent experiments) and Western blot analysis (right) confirmed the effectiveness of *shPRR11* silencing in ACHN cells. **(B)** Tumor weight statistic for mice injected subcutaneously with ACHN cells transfected with *shNC* or *shPRR11* and treated with or without SC79 (n = 8 per group). **(C)** Representative images of IHC staining of tumor tissue from each group of mice in the xenograft model. **(D)** Gross diagram of tail vein lung metastasis from mice injected ACHN cells transfected with *shNC* or *shPRR11* with or without SC79 treatment (left). Amount of lung surface tumors in each group was statistically analyzed (right, n = 3 per group). **(E)** Schematic anatomy of the popliteal lymph node metastasis model. **(F)** Representative images of H&E staining of popliteal lymph nodes from each group. L = normal lymphoid tissue; T = metastatic tumor. The *p*-values were calculated with two-tailed Student's t-test (A) and one-way ANOVA with Tukey's multiple comparisons test (B, D). Data are presented as mean values ± SD.

Supplementary Tables

Table S1. Detailed information on protein molecular docking interaction sites.

FBXW7- PRR11 interface residue pair(s)	Hydrogen Bonds(Å)
Val 8-Asn 283	3.843
Gly 9-Val 281	4.257
Gly 9-Asn 283	2.564
Ser 10-Asn 283	4.644
Ser 10-Leu 285	4.433
Lys 11-Thr 279	2.902
Lys 11-Arg 280	4.08
Gln 43-Leu 339	3.825
Gln 44-Arg 335	2.392
Gln 44-Gln 338	4.553
Gln 44-Leu 339	3.678
Gln 47-Arg 335	3.75
Gln 47-Gln 338	2.557
Gln 47-Leu 339	3.757
Gln 48-Gln 331	2.736
Gln 48-Arg 335	2.547
Gln 48-Gln 338	4.059
Glu 50-Arg 343	4.213
Glu 51-Arg 334	2.552
Glu 51-Gln 338	2.285
Glu 51-Arg 343	4.613
His 52-Glu 320	4.135
Ala 54-Arg 343	4.106
Arg 55-Glu 320	3.299
Arg 55-Arg 334	4.543
Gly 499-Val 328	4.098
Gly 499-Met 329	4.592
Gly 499-Ala 332	4.045
Val 501-Val 328	4.998
Val 501-Ala 332	2.758
Val 501-Arg 335	4.133
Asp 520-Val 328	4.973
Met 522-Val 328	3.362
Met 522-Gln 331	4.285
Lys 524-Thr 326	4.262
Lys 524-Val 328	2.781
Lys 524-Met 329	3.733
Cys 533-Thr 326	3.285
Cys 533-Met 329	3.786
Leu 534-Thr 326	3.547
His 535-Thr 326	3.491
Thr 536-Thr 326	3.018
Thr 536-Pro 327	4.837

FBXW7- PRR11 interface residue pair(s)	Hydrogen Bonds(Å)
Thr 536-Val 328	1.493
Thr 536-Met 329	4.42
Leu 537-Val 328	4.986
Gln 538-Thr 314	4.352
Gln 538-Glu 317	3.2
Gln 538-Pro 327	4.103
Gln 538-Val 328	3.312
Gln 538-Gln 331	4.895
Gly 539-Thr 314	4.023
Gly 539-Glu 317	4.967
Arg 564-Thr 311	3.934
Arg 564-Pro 312	3.347
Arg 564-Thr 314	3.869
Cys 573-Thr 311	2.928
Ile 574-Ser 307	4.837
Ile 574-Gly 309	3.791
Ile 574-Thr 311	2.272
His 575-Gly 309	2.669
His 575-Gly 310	4.529
His 575-Thr 311	3.01
His 575-Pro 312	4.88
Thr 576-Arg 306	3.358
Thr 576-Gly 310	3.24
Thr 576-Thr 311	3.276
Thr 576-Pro 312	1.717
Thr 578-Arg 306	3.339
Gly 579-Leu 299	3.329
His 580-Leu 299	4.729
Gln 581-Leu 298	3.77
Gln 581-Leu 299	3.849
Ser 601-Val 284	4.978
Gln 618-Leu 295	3.131
Gly 619-Pro 288	2.64
Pro 620-Thr 287	4.571
Pro 620-Pro 288	3.849
Lys 622-Val 284	4.436
Lys 622-Ile 286	3.147
Lys 622-Thr 287	4.34
Lys 622-Pro 288	3.461
Gln 624-Leu 285	2.543
Gln 624-Ile 286	4.398
Gln 624-Thr 287	2.655
Gln 624-Pro 288	4.524
Ser 625-Leu 285	4.553

FBXW7-PRR11 (Docking Score：-266.11, Confidence Score：0.9107, Ligand RMSD：39.11

Table S2. Clinicopathological characteristics of the HKidE180Su02 cohort on PRR11.

	Variables	Total (n=149)	PRR11 high expression (n=75)	PRR11 low expression (n=74)	*p*-value	Statistics method
Gender (%)	female	42 (28.19)	19 (25.33)	23(31.08)	0.4356	Chi-square
	male	107 (71.81)	56 (74.67)	51 (68.92)		
Age (year) (%)	<=65	119 (79.87)	57 (76.00)	62 (83.78)	0.2361	Chi-square
	>65	30 (20.13)	18 (24.00)	12 (16.22)		
Grade (%)	<Grade III	102 (68.46)	44 (58.67)	58 (78.38)	**0.0096**	Chi-square
	>= Grade III	47 (31.54)	31 (41.33)	16 (21.62)		
AJCC stage (%)	<Stage II	121 (81.21)	56 (74.67)	65 (87.84)	**0.0396**	Chi-square
	>=Stage II	28 (18.79)	19 (25.33)	9 (12.16)		
N (%)	N>=1	3 (2.01)	3 (4.00)	0 (0)	0.2450	Fisher’s exact
	N0	146 (97.99)	72 (96.00)	74 (100.00)		
Tumor Size (%)	<=4 cm	74 (49.66)	35 (46.67)	39 (52.70)	0.4612	Chi-square
	>4 cm	75 (50.34)	40 (53.33)	35 (47.30)		

PRR11 expression group: The median of the PRR11 average optical density was cut-off value;

Tumor size: The longest diameter, cm;

Statistical significance was determined by two-tailed Chi-square or two-tailed Fisher’s exact test. No adjustments were made for multiple comparisons.

Table S3. Clinicopathological characteristics of the HKidE180Su02 cohort on FBXW7.

	Variables	Total (n=149)	FBXW7 high expression (n=75)	FBXW7 low expression (n=74)	*p*-value	Statistics method
Gender (%)	female	42 (28.19)	23(30.67)	19(25.68)	0.4984	Chi-square
	male	107 (71.81)	52(69.33)	55(74.32)		
Age (year) (%)	<=65	119 (79.87)	64 (85.33)	55 (74.32)	0.0938	Chi-square
	>65	30 (20.13)	11 (14.67)	19(25.68)		
Grade (%)	<Grade III	102 (68.46)	52 (69.33)	50 (67.57)	0.8166	Chi-square
	>= Grade III	47 (31.54)	23 (30.67)	24 (32.43)		
AJCC stage (%)	<Stage II	121 (81.21)	63 (84.00)	58 (78.38)	0.3798	Chi-square
	>=Stage II	28 (18.79)	12 (16.00)	16 (21.62)		
N (%)	N>=1	3 (2.01)	0 (0)	3 (4.05)	0.1200	Fisher's exact
	N0	146 (97.99)	75 (100.00)	71 (95.95)		
Tumor Size (%)	<=4 cm	74 (49.66)	43 (57.33)	31 (41.89)	0.0594	Chi-square
	>4 cm	75 (50.34)	32 (42.67)	43 (58.11)		

FBXW7 expression group: The median of the FBXW7 average optical density was cut-off value;

Tumor size: The longest diameter, cm;

Statistical significance was determined by two-tailed Chi-square or two-tailed Fisher's exact test. No adjustments were made for multiple comparisons.

Table S4. GO analysis of PRR11-related genes in the TCGA cohort.

Ontology	ID	Description	Count	*p*-value	*p*-adjust	*q*-value
BP	GO:0007059	Chromosome segregation	28	3.48E-16	8.17E-13	7.09E-13
BP	GO:0098813	Nuclear chromosome segregation	24	1.83E-15	1.96E-12	1.70E-12
BP	GO:0000819	Sister chromatid segregation	21	2.50E-15	1.96E-12	1.70E-12
BP	GO:0000280	Nuclear division	27	7.94E-15	4.66E-12	4.04E-12
BP	GO:0048285	Organelle fission	27	9.19E-14	4.25E-11	3.69E-11
BP	GO:0000070	Mitotic sister chromatid segregation	18	1.20E-13	4.25E-11	3.69E-11
BP	GO:0140014	Mitotic nuclear division	21	1.27E-13	4.25E-11	3.69E-11
BP	GO:0006310	DNA recombination	20	6.35E-11	1.86E-08	1.62E-08
BP	GO:0006261	DNA-templated DNA replication	14	3.44E-10	8.12E-08	7.05E-08
BP	GO:0045787	Positive regulation of cell cycle	19	3.46E-10	8.12E-08	7.05E-08
CC	GO:0000228	Nuclear chromosome	17	2.78E-11	7.89E-09	5.94E-09
CC	GO:0000793	Condensed chromosome	18	8.76E-11	1.24E-08	9.36E-09
CC	GO:0005819	Spindle	21	3.55E-10	3.36E-08	2.53E-08
CC	GO:0098687	Chromosomal region	18	2.28E-08	1.62E-06	1.22E-06
CC	GO:0000794	Condensed nuclear chromosome	9	4.74E-08	2.69E-06	2.03E-06
CC	GO:0000922	Spindle pole	12	6.83E-08	3.23E-06	2.43E-06
CC	GO:0043596	Nuclear replication fork	6	5.34E-07	1.95E-05	1.47E-05
CC	GO:0005635	Nuclear envelope	18	5.49E-07	1.95E-05	1.47E-05
CC	GO:0035861	Site of double-strand break	8	9.83E-07	3.10E-05	2.33E-05
CC	GO:0090734	Site of DNA damage	9	1.14E-06	3.23E-05	2.43E-05
MF	GO:0106310	Protein serine kinase activity	18	9.35E-09	3.39E-06	2.62E-06
MF	GO:0140097	Catalytic activity, acting on DNA	14	9.81E-08	1.46E-05	1.12E-05
MF	GO:0004674	Protein serine/threonine kinase activity	18	1.20E-07	1.46E-05	1.12E-05
MF	GO:0016887	ATP hydrolysis activity	17	3.72E-07	3.37E-05	2.60E-05

Ontology	ID	Description	Count	*p*-value	*p*-adjust	*q*-value
MF	GO:0003777	Microtubule motor activity	7	3.46E-06	0.000251	0.000194
MF	GO:0140297	DNA-binding transcription factor binding	16	1.18E-05	0.000654	0.000504
MF	GO:0008574	Pus-end-directed microtubule motor activity	4	1.26E-05	0.000654	0.000504
MF	GO:0003697	Single-stranded DNA binding	8	1.59E-05	0.000719	0.000555
MF	GO:0008094	ATP-dependent activity, acting on DNA	8	2.02E-05	0.000815	0.000629
MF	GO:0042393	Histone binding	11	2.75E-05	0.000998	0.000770

BP: Biological process;

MF: Molecular function;

CC: Cellular component;

The statistical significance of the GO analysis was determined by two-tailed Fisher’s exact test.

Table S5. KEGG analysis of PRR11-related genes in the TCGA cohort.

ID	Description	Count	*p*-value	*p*-adjust	*q*-value
hsa03440	Homologous recombination	6	4.60E-06	0.000750	0.000717
hsa03030	DNA replication	4	0.000587	0.047863	0.045745
hsa04110	Cell cycle	7	0.001538	0.076313	0.072937
hsa03430	Mismatch repair	3	0.001873	0.076313	0.072937
hsa03460	Fanconi anemia pathway	4	0.002717	0.077198	0.073784
hsa05010	Alzheimer disease	11	0.002842	0.077198	0.073784
hsa04120	Ubiquitin mediated proteolysis	6	0.004361	0.088517	0.084602
hsa03250	Viral life cycle - HIV-1	4	0.004753	0.088517	0.084602
hsa04814	Motor proteins	7	0.004887	0.088517	0.084602
hsa05203	Viral carcinogenesis	7	0.006585	0.101689	0.097191

The statistical significance of the KEGG analysis was determined by two-tailed Fisher's exact test.

Table S6. GSEA of PRR11-related genes based on RNA-seq.

Description	Set size	NES	*p*-value	*p*-adjust	*q*-value
O glycan biosynthesis	19	-1.983475	0.001908	0.073573	0.065428
Basal cell carcinoma	32	-1.938257	0.001905	0.073573	0.065428
DNA replication	35	1.871137	0.002110	0.073573	0.065428
Huntington disease	152	1.673846	0.002096	0.073573	0.065428
Oxidative phosphorylation	102	1.615537	0.002114	0.073573	0.065428
MAPK signaling pathway	191	-1.461674	0.003617	0.104882	0.093271
Nucleotide excision repair	43	1.638751	0.006383	0.138830	0.123460
WNT signaling pathway	111	-1.578611	0.005650	0.138830	0.123460
Cell cycle	117	1.469710	0.008753	0.169220	0.150486
Parkinson disease	100	1.530321	0.010460	0.182008	0.161859
Homologous recombination	25	1.668655	0.020833	0.211188	0.187808
Mismatch repair	22	1.621682	0.021645	0.211188	0.187808

NES: Normalized enrichment score;

The statistical significance of the GSEA results was determined by two-tailed Fisher's exact test.

Table S7. PRR11-related KEGG analysis based on RNA-seq.

ID	Description	Count	*p*-value	*p*-adjust	*q*-value
hsa04820	Cytoskeleton in muscle cells	9	0.001201	0.133322	0.127064
hsa05165	Human papillomavirus infection	9	0.012791	0.402801	0.383892
hsa04151	PI3K-Akt signaling pathway	9	0.020940	0.402801	0.383892
hsa04630	JAK-STAT signaling pathway	6	0.011766	0.402801	0.383892
hsa04510	Focal adhesion	6	0.027213	0.402801	0.383892
hsa04380	Osteoclast differentiation	5	0.022150	0.402801	0.383892
hsa04115	p53 signaling pathway	4	0.010118	0.402801	0.383892
hsa01232	Nucleotide metabolism	4	0.015478	0.402801	0.383892
hsa05412	Arrhythmogenic right ventricular cardiomyopathy	4	0.016096	0.402801	0.383892
hsa04512	ECM-receptor interaction	4	0.018041	0.402801	0.383892
hsa04750	Inflammatory mediator regulation of TRP channels	4	0.025554	0.402801	0.383892
hsa05410	Hypertrophic cardiomyopathy	4	0.025554	0.402801	0.383892
hsa00730	Thiamine metabolism	3	0.000577	0.128205	0.122187
hsa04930	Type II diabetes mellitus	3	0.015856	0.402801	0.383892
hsa00240	Pyrimidine metabolism	3	0.027614	0.402801	0.383892

The statistical significance of the KEGG analysis was determined by two-tailed Fisher's exact test.

Table S8. Details of the antibodies used in this study.

Antibody	Catalog No.	Source	Dilution or amount
Flag-tag	F1804	Sigma	IP/1 μg WB/1:1000 IF/1:200
HA-tag	TA180128	Origene	IP/1 μg WB/1:1000
HA-tag	AE105	ABclonal	IF/1:200 WB/1:10000
Myc-tag	AE010	ABclonal	IP/1 μg WB/1:1000
His-tag	10001-0-AP	Proteintech	WB/1:1000
GST-tag	10000-0-AP	Proteintech	WB/1:1000
PRR11	PA5-118199	Invitrogen	IP/1 μg WB/1:1000
PRR11	CSB-PA836225LA01HU	Cusabio	IHC/1:200
FBXW7	A301-720A	Invitrogen	IP/1 μg WB/1:1000
GSK3β	12456	Cell Signaling Technology	IP/1 μg WB/1:1000
GSK3β	67329-1-Ig	Proteintech	WB/1:5000
GAPDH	60004-1-Ig	Proteintech	WB/1:10000
Ubiquitin	ab7254	Abcam	WB/1:1000
Cleaved PARP	5625	Cell Signaling Technology	WB/1:1000
p-CHK2 (T68)	ab32148	Abcam	WB/1:1000
γ-H2AX (S139)	ab81299	Abcam	WB/1:2000 IHC/1:200 IF/1:200
SOD2	ab13533	Abcam	WB/1:2000
mTOR	ab32028	Abcam	WB/1:1000
p-mTOR (S2448)	ab109268	Abcam	WB/1:1000
AKT	4691	Cell Signaling Technology	WB/1:1000
p-AKT (T308)	9275	Cell Signaling Technology	WB/1:1000

Antibody	Catalog No.	Source	Dilution or amount
p-AKT (S473)	4060	Cell Signaling Technology	WB/1:1000 IHC/1:200
p-GSK3β (S9)	5558	Cell Signaling Technology	WB/1:1000
Ki67	ab16667	Abcam	IHC/1:200
Thiophosphate ester	ab92570	Abcam	WB/1:5000

IP: immunoprecipitation; WB: Western blot; IF: Immunofluorescence; IHC: Immunohistochemistry.

Table S9. Primers used for qRT-PCR.

Gene	Forward primer (5'-3')	Reverse primer (5'-3')
PRR11	GAAGCTGGCTAACATCATCCTG	CTCTGGGTTATGCAGTTCTGG
FBXW7	CCACTGGGCTTGTACCATGTT	CAGATGTAATTCGGCGTCGTT
GSK3β	GGCAGCATGAAAGTTAGCAGA	GGCGACCAGTTCTCCTGAATC
GAPDH	GGAGCGAGATCCCTCCAAAAT	GGCTGTTGTCATACTTCTCATGG

Description of Supplementary Datasets S1-S5

Dataset S1. Immunoprecipitation-mass spectrometry (IP-MS) results for PRR11.

Sheet “protein” describes key information such as “Protein ID”, “Score”, “Sequence coverage (%)”, “Peptides” and “Intensity”. Sheet “peptide” describes key information such as “Sequence”, “Mass”, “PEP”, “Score” and “Intensity”.

Dataset S2. PRR11-related genes based on the TCGA-KIRC cohort (top 200).

Sheet contains “SYMBOL”, “COR”, “p-value” and “FDR” parameters for PRR11-related genes.

Dataset S3. GO and KEGG analysis of PRR11-related genes in TCGA-KIRC cohort.

Sheet “GO” describes key information such as “Description”, “pvalue”, “p.adjust”, “qvalue”, “gene ID” and “Count”. Sheet “KEGG” describes key information such as “Description”, “pvalue”, “p.adjust”, “qvalue”, “gene ID” and “Count”.

Dataset S4. GSEA of PRR11-related genes based on RNA-seq.

Sheet contains “Description”, “NES”, “p-value”, “p.adjust”, “qvalue”, “rank” and “gene ID” parameters.

Dataset S5. PRR11-related KEGG analysis based on RNA-seq.

Sheet contains “Description”, “Gene Ratio”, “p-value”, “p.adjust”, “qvalue”, “rank” and “gene ID” parameters.
